# Efficacy, Immunogenicity, Safety, and Evidence Independence of CYD-TDV and TAK-003 Dengue Vaccines in Children and Adolescents: A Systematic Review and Meta-Analysis

**DOI:** 10.3390/vaccines14100836

**Published:** 2026-09-22

**Authors:** Jessica P. Sanchez-Guanilo, Carmen A. Contreras, Miguel A. Arce-Huamani

**Affiliations:** 1 Programa de Estudio de Medicina Humana, Facultad de Medicina Humana, Universidad Privada Antenor Orrego, Trujillo 13008, Peru; pattysg94@gmail.com (J.P.S.-G.); carmencontrerasg@gmail.com (C.A.C.); 2 Programa Academico de Medicina Humana, Facultad de Ciencias de la Salud, Universidad Privada Norbert Wiener, Lima 15072, Peru

**Keywords:** dengue, CYD-TDV, TAK-003, children, adolescents, vaccine efficacy, immunogenicity

## Abstract

**Background/Objectives**: The efficacy and safety profiles of CYD-TDV and TAK-003 are established, but multiple publications may derive from the same randomized trial population and therefore not represent independent evidence. We evaluated efficacy, immunogenicity, and safety while distinguishing independent trials from overlapping follow-up reports and interpreting absolute effects within each programme. **Methods**: We conducted a PRISMA 2020 systematic review and meta-analysis of randomized trials and follow-up reports from five databases. Outcomes included virologically confirmed dengue, hospitalization, severe dengue, serotype-specific efficacy, immunogenicity, and safety. Risk ratios (RRs) with 95% confidence intervals (CIs) were calculated; immunogenicity and safety were synthesized narratively. RoB 2 and GRADE were applied. **Results**: Nine reports were included. CYD-TDV reduced virologically confirmed dengue (RR = 0.41; 95% CI: 0.35–0.48) and hospitalization (RR = 0.41; 95% CI: 0.25–0.67). Across two independent CYD-TDV trials, absolute reductions were 23.2 virologically confirmed dengue cases per 1000 participants (NNV = 44) and 12.0–50.0 hospitalizations per 1000 (NNVs = 84–20). In the primary TIDES report, TAK-003 reduced virologically confirmed dengue (RR = 0.28; 95% CI: 0.22–0.35) and hospitalization (RR = 0.10; 95% CI: 0.05–0.18), with absolute reductions of 23.6 and 12.9 per 1000 (NNVs = 43 and 78). Later TIDES reports extended follow-up of the same population. **Conclusions**: Both vaccines reduced dengue outcomes within their respective trial programmes. Absolute effects should be interpreted according to baseline risk and trial context, not as direct between-vaccine comparisons. The synthesis distinguishes extended follow-up from independent replication.

## 1. Introduction

Dengue remains a major and expanding public health threat, with a growing burden among children and adolescents [1,2]. Transmission intensity varies markedly across endemic settings because of differences in force of infection, population seroprevalence, and age-specific exposure patterns [3,4]. Climate-sensitive changes may further modify the geographical distribution and intensity of dengue transmission [5]. In pediatric populations, disease severity also varies according to infecting serotype and previous immune status [6]. These factors are directly relevant to vaccination because vaccine performance depends on the product administered, baseline dengue serostatus, age, circulating serotypes, and duration of follow-up.

Placebo-controlled trials have shown that TAK-003 reduces virologically confirmed and hospitalized dengue in children and adolescents [7,8]. Longer-term analyses demonstrated sustained protection against hospitalization, although efficacy against symptomatic dengue declined over time and varied according to baseline serostatus and infecting serotype [9,10]. Age-stratified analyses and recent pooled evidence have also supported its immunogenicity and efficacy across pediatric and adolescent populations while confirming relevant variation between subgroups [11,12].

For CYD-TDV, the benefit–risk profile is strongly determined by previous dengue exposure. Efficacy is more favorable among baseline-seropositive recipients, whereas protection is limited or uncertain among seronegative individuals [13,14]. Long-term follow-up has shown sustained protection against hospitalized and severe dengue among seropositive participants, particularly those aged ≥9 years [15]. These findings also raise important safety concerns regarding the vaccination of individuals without evidence of previous dengue infection.

Previous evidence syntheses have extensively evaluated the efficacy, immunogenicity, and safety of dengue vaccines. Vaccine-specific systematic reviews have addressed CYD-TDV and TAK-003 separately [12,16,17,18,19,20], while other reviews have examined both vaccines or broader dengue vaccine portfolios across pediatric, adolescent, and general populations [21,22,23,24,25,26]. Additional publications have also addressed vaccine development, implementation, and programmatic considerations [27]. Accordingly, the present review was not designed to establish a previously unrecognized vaccine effect or to rank CYD-TDV against TAK-003. Rather, it addresses a narrower methodological and clinical question concerning how the placebo-controlled evidence in children and adolescents is structured and interpreted. Specifically, we linked successive publications to their source randomized trial populations, distinguished extended follow-up from independent replication, avoided treating overlapping reports as independent evidence, and interpreted relative and absolute effects within each vaccine programme and its corresponding baseline risk. We also integrated these findings with outcome-specific risk-of-bias and certainty assessments and with long-term safety evidence according to baseline serostatus when available. A broader comparison with previous evidence syntheses is provided in Section 4 and Supplementary Table S10. These analyses are intended to refine the interpretation and strength of the existing evidence rather than to imply a direct comparison between the two vaccines.

The current programmatic relevance of CYD-TDV has also changed. The manufacturer has announced the discontinuation of Dengvaxia production because of limited global demand [28]. Although this limits its future availability for vaccination programmes, the CYD-TDV evidence remains scientifically and clinically relevant because it provides the clearest example of how baseline dengue serostatus can alter both vaccine benefit and the subsequent risk of hospitalized or severe dengue. Its long-term safety findings therefore remain important for interpreting dengue vaccine biology, regulatory decisions, pre-vaccination screening policies, and the development of future vaccine products.

Accordingly, the prespecified objective of this systematic review and meta-analysis was to synthesize placebo-controlled evidence on the efficacy, immunogenicity, and safety of CYD-TDV and TAK-003 in children and adolescents, including virologically confirmed dengue, dengue-related hospitalization, severe dengue, serotype-specific efficacy, immunogenicity, and safety. Because these vaccine effects have already been extensively addressed in previous reviews, the present synthesis additionally focused on how the underlying evidence should be interpreted. Specifically, we identified the independent randomized trial populations supporting each vaccine programme, distinguished extended follow-up from independent replication, avoided treating overlapping reports as statistically independent evidence, and interpreted relative and absolute effects within their corresponding trial populations and baseline-risk contexts. Long-term hospitalized and severe dengue outcomes were also examined according to baseline serostatus and age when reported. These analyses were intended to clarify the strength, independence, and clinical applicability of the existing evidence rather than to establish a head-to-head comparison between CYD-TDV and TAK-003.

## 2. Materials and Methods

### 2.1. Study Design and Reporting Framework

We conducted a systematic review and meta-analysis to evaluate the efficacy, immunogenicity, and safety of the CYD-TDV and TAK-003 dengue vaccines in children and adolescents. Clinically relevant outcomes included virologically confirmed dengue, dengue-related hospitalization, severe dengue, serotype-specific efficacy, immunogenicity, and short- and long-term safety. Eligibility was restricted to randomized placebo-controlled trials and related follow-up reports because a concurrent placebo comparator was prespecified in the review protocol. The placebo comparator was therefore treated as an eligibility and trial-design characteristic rather than as a feature distinguishing the present review from previous evidence syntheses. The manuscript was prepared in accordance with the Preferred Reporting Items for Systematic Reviews and Meta-Analyses (PRISMA) 2020 statement. The PRISMA 2020 flow diagram is presented in the main text as Figure 1, and the completed PRISMA 2020 checklist is provided as a separate file for editorial assessment. The review protocol was registered in PROSPERO under registration number CRD42024580531.

### 2.2. Eligibility Criteria

Studies were eligible if they met the following criteria: randomized clinical trial or related placebo-controlled follow-up report; evaluation of CYD-TDV or TAK-003 dengue vaccines; inclusion of children and/or adolescents; comparison with placebo; and reporting of at least one relevant outcome, including virologically confirmed dengue, dengue-related hospitalization, severe dengue, serotype-specific efficacy, immunogenicity, or safety. Long-term, post hoc, or secondary follow-up reports derived from randomized dengue vaccine trial programmes were also eligible when they provided additional outcome-specific evidence on severe dengue, dengue-related hospitalization, baseline serostatus, age-related effects, or long-term safety. Follow-up reports involving previously described trial populations were retained for narrative, subgroup, certainty, or safety assessments but were not treated as statistically independent studies in pooled analyses. Reports combining phase III populations with a related randomized trial cohort were used only for narrative long-term safety and subgroup synthesis.

Studies were excluded if they were narrative reviews, letters to the editor, case reports, case series, conference abstracts, books, animal studies, in vitro studies, studies without published or completed results, or trials that did not evaluate the target population. Studies without a concurrent placebo group were also excluded, including single-arm studies, open-label studies without a control group, observational effectiveness studies, and randomized trials using only an active vaccine or another intervention as the comparator. Reports with overlapping trial populations were not automatically excluded; however, when they represented repeated analyses of the same participants at different follow-up periods, they were handled descriptively and were not treated as statistically independent in the main pooled analyses.

The requirement for a concurrent placebo comparator was prespecified in the review protocol and was retained to maintain consistency with the original eligibility criteria. This restriction was not intended to distinguish the present review from previous dengue vaccine reviews or to imply that placebo-controlled evidence was unique to this synthesis. Studies without a concurrent placebo group, including coadministration and active-comparator studies, addressed different clinical questions and were therefore outside the prespecified scope of the review [29,30].

### 2.3. Information Sources and Search Strategy

A comprehensive literature search was conducted in PubMed, Web of Science, Scopus, Cochrane, and Embase. The search strategy combined terms related to dengue, dengue virus, dengue vaccines, CYD-TDV, TAK-003, tetravalent dengue vaccine, randomized trials, controlled clinical trials, children, adolescents, efficacy, immunogenicity, and safety. Medical Subject Headings and Boolean operators were used when applicable according to each database.

No language restriction was applied. The full search strategy used for each database is provided in the Supplementary Materials.

### 2.4. Study Selection Process

All identified records were imported into Rayyan for study screening and selection. Duplicate records were removed before screening. Titles and abstracts were screened according to the predefined eligibility criteria. Potentially eligible reports were then assessed in full text. No automation tool was used to exclude records.

The search identified 1462 records from five databases: Cochrane (*n* = 210), Embase (*n* = 82), PubMed (*n* = 133), Scopus (*n* = 828), and Web of Science (*n* = 209). After removing 404 duplicate records, 1058 unique records were screened by title and abstract. Of these, 1042 records were excluded. Sixteen full-text reports were assessed for eligibility. Seven reports were excluded: three because they represented overlapping populations with shorter follow-up or provided no additional relevant outcome information, two because they did not evaluate the target population, and two because they did not meet the inclusion criteria. Overall, nine randomized trial and related placebo-controlled follow-up reports were included in the systematic review. The study selection process is shown in the PRISMA 2020 flow diagram (Figure 1).

### 2.5. Data Extraction

Data extraction was performed independently by two reviewers using separate copies of a standardized Microsoft Excel 2019 form. After completion, the extracted datasets were compared field by field. Discrepancies were resolved through discussion and consensus; when disagreement persisted, a third reviewer adjudicated the final decision. No automation tool was used during data extraction. Because the extraction form contained a combination of categorical, numerical, and verbatim fields, a formal inter-reviewer reliability coefficient, such as Cohen’s kappa, was not calculated. Instead, agreement was verified through direct field-by-field comparison, and all discrepancies were resolved before the data were entered into the quantitative or narrative synthesis.

The extracted information included author, publication year, country or setting, study design, participant characteristics, sample size, age range, vaccine type, vaccine dose, route of administration, number of doses, vaccination schedule, comparator, follow-up duration, baseline serostatus when available, virologically confirmed dengue, serotype-specific outcomes, immunogenicity measures, dengue-related hospitalization, severe dengue, and safety outcomes. For safety, we extracted overall serious adverse events, vaccine-related serious adverse events, severe dengue occurring after vaccination, deaths, withdrawals or study discontinuations due to adverse events, and the duration of safety follow-up. Long-term safety outcomes stratified by baseline serostatus were also extracted when reported.

For efficacy and hospitalization outcomes, the number of events and total participants in the vaccine and placebo groups were extracted whenever available. Where reported, subgroup data were also extracted according to baseline dengue serostatus, age group, and the occurrence of dengue-related hospitalization. For immunogenicity, antibody responses were extracted according to the metric reported in each study, including geometric mean titres, geometric mean ratios, log-transformed titres, seropositivity rates, and PRNT50-based neutralizing antibody measures. Data were recorded separately by vaccine, dengue virus serotype, baseline serostatus, and follow-up time point.

### 2.6. Outcomes

The primary outcome was virologically confirmed dengue among vaccine recipients compared with placebo recipients. Secondary outcomes included dengue-related hospitalization, severe dengue, serotype-specific efficacy against DENV-1, DENV-2, DENV-3, and DENV-4, immunogenicity, and safety.

Serotype-specific efficacy was analyzed separately for each dengue virus serotype. No single cross-serotype pooled estimate was considered a primary outcome because serotype-specific outcomes within the same trial population are correlated and may generate a unit-of-analysis problem.

Immunogenicity was considered a supportive outcome because antibody responses were reported using titre-based measures that varied across vaccine type, serotype, baseline serostatus, and follow-up time point.

Safety outcomes included serious adverse events, vaccine-related serious adverse events, deaths, withdrawals or study discontinuations due to adverse events, and clinically relevant long-term safety outcomes. Hospitalized or severe dengue occurring after vaccination was additionally considered a safety outcome when reported according to baseline serostatus, particularly for CYD-TDV.

### 2.7. Risk-of-Bias Assessment

Risk of bias was assessed using the revised Cochrane Risk of Bias tool for randomized trials (RoB 2; version 22 August 2019), which is appropriate for individually randomized, parallel-group trials. The assessment focused on the effect of assignment to the intervention and covered five domains: bias arising from the randomization process, bias due to deviations from the intended interventions, bias due to missing outcome data, bias in the measurement of the outcome, and bias in the selection of the reported result.

Risk-of-bias assessments were outcome-specific. When a trial report contributed information to more than one outcome, separate judgements were made for virologically confirmed dengue, dengue-related hospitalization, severe dengue, immunogenicity, and safety, as applicable. Signalling questions and domain-level algorithms were used to classify each result as having low risk of bias, some concerns, or high risk of bias. An overall judgement was then assigned according to the RoB 2 guidance.

Two reviewers independently completed the assessments. Disagreements were resolved through discussion and consensus, and a third reviewer was consulted when necessary. Sponsor or manufacturer involvement was recorded separately as a contextual funding consideration but was not treated as an additional RoB 2 domain or as an automatic reason for assigning a high risk-of-bias judgement. No automation tool was used to make the final risk-of-bias judgements.

### 2.8. GRADE Assessment

The certainty of the evidence for each prespecified major outcome was assessed using the Grading of Recommendations Assessment, Development and Evaluation (GRADE) approach. Separate assessments were conducted for CYD-TDV and TAK-003 because the two vaccines were evaluated in different clinical trial programmes and were not directly compared. The outcomes assessed were virologically confirmed dengue, dengue-related hospitalization, severe dengue, immunogenicity, and safety.

Evidence from randomized trials was initially rated as high certainty. Certainty was downgraded by one or two levels when a domain was judged to present serious or very serious concerns regarding risk of bias, inconsistency, indirectness, imprecision, or publication bias. When an outcome was informed by only one independent trial or by overlapping follow-up reports from the same trial programme, inconsistency was considered not assessable. Publication bias was evaluated qualitatively because the limited number of independent studies precluded formal statistical assessment. Two reviewers independently performed the GRADE assessments. Disagreements were resolved by consensus, with consultation of a third reviewer when necessary. Final ratings were categorized as high, moderate, low, or very low certainty.

### 2.9. Data Synthesis and Statistical Analysis

The findings were summarized descriptively and quantitatively. For dichotomous outcomes, including virologically confirmed dengue and dengue-related hospitalization, risk ratios (RRs) with 95% confidence intervals (CIs) were calculated. Immunogenicity outcomes were not pooled because the included reports used different measurement scales and summary metrics, including geometric mean titres, geometric mean ratios, log-transformed titres, seropositivity rates, and PRNT50-based neutralization measures.

To improve the clinical interpretation of the main efficacy outcomes, baseline risks, absolute risk reductions (ARRs), and numbers needed to vaccinate (NNVs) were calculated from the reported event counts and denominators. Baseline risk was defined as the event proportion in the placebo group, and ARR was calculated as the difference between the placebo-group and vaccine-group risks. Ninety-five percent confidence intervals for the absolute risk differences were calculated using the Newcombe method based on Wilson score intervals for two independent proportions. The NNV was calculated as the reciprocal of the ARR and rounded up to the nearest whole number; its 95% CI was obtained by inverting the corresponding confidence limits of the ARR when both limits indicated benefit. For CYD-TDV, absolute effects against virologically confirmed dengue were calculated using the combined event counts from the two independent efficacy trials. Because hospitalization risks and endpoint-specific denominators differed substantially between the CYD-TDV trials, absolute hospitalization effects were calculated separately for each trial rather than combined into a single NNV. For TAK-003, the primary efficacy report was used for the main absolute-effect estimates to avoid double counting participants from overlapping follow-up publications. Absolute effects from subsequent follow-up reports were considered descriptive and were not treated as independent estimates.

Statistical heterogeneity was assessed using the I^2^ statistic and the Cochran Q test. Heterogeneity was considered relevant when the *p* value for heterogeneity was ≤0.10 and I^2^ was greater than 40%. When pooling was appropriate, a random-effects model was used because clinical and methodological variability was expected across trials. Potential sources of heterogeneity were explored qualitatively by comparing geographic and epidemiological settings, participant age distribution, baseline dengue serostatus, circulating serotypes, surveillance procedures, endpoint-specific denominators, baseline event risks, and duration of follow-up. Formal subgroup analyses or meta-regression were not performed when fewer than three independent studies contributed to an outcome because such analyses would not provide reliable estimates of between-study effect modification. A formal leave-one-out sensitivity analysis was also not considered informative for the CYD-TDV hospitalization outcome because only two independent trials contributed to the pooled estimate; excluding either trial would leave a single study and would therefore not permit evaluation of pooled heterogeneity.

For CYD-TDV, the main meta-analysis included the two eligible independent placebo-controlled efficacy trials, CYD14 and CYD15. These trials were not selected as a subset of the available evidence; rather, they represented the independent trial populations meeting the prespecified eligibility criteria and contributing the relevant efficacy outcomes. Pooled estimates were calculated for virologically confirmed dengue, dengue-related hospitalization, and serotype-specific efficacy. Subsequent long-term reports that included participants from these trials were retained for subgroup, severe dengue, and safety analyses but were not treated as additional independent efficacy studies.

For TAK-003, the primary TIDES efficacy report was used as the main estimate for virologically confirmed dengue and dengue-related hospitalization because it represented the principal independent randomized trial population contributing these efficacy outcomes. Extended TIDES follow-up reports were summarized descriptively and graphically but were not pooled with the primary report because they included overlapping participants from the same clinical trial programme and evaluated different follow-up periods. This approach was used to avoid double counting participants and to distinguish longer observation of the same randomized population from independent replication of the efficacy findings.

Immunogenicity findings were synthesized narratively by vaccine, serotype, baseline serostatus, and follow-up time point. Study-specific estimates were retained using the metric originally reported by each trial. Raw mean differences were not pooled because antibody responses were expressed using non-equivalent scales and transformations. Standardized mean differences were also not calculated because differences in assay methods, titre transformations, time points, and biological interpretation would have limited their clinical meaning. Immunogenicity was therefore treated as supportive evidence rather than as a directly comparable pooled efficacy endpoint.

Where data permitted, we sought to examine vaccine effects according to baseline dengue serostatus (seropositive versus seronegative), age group (≥9 versus <9 years), and whether the effect on dengue-related hospitalization differed according to baseline serostatus. A formal subgroup meta-analysis or interaction analysis was not performed because these strata were not reported consistently across the included trial reports, subgroup definitions and follow-up periods differed, and several subgroup estimates were derived from overlapping analyses of the same trial populations. These findings were therefore summarized narratively and interpreted as exploratory rather than as statistically independent comparative evidence.

Safety outcomes were synthesized narratively by vaccine and follow-up period. A pooled safety estimate was not calculated because adverse-event definitions, reporting periods, denominators, and causal attribution to vaccination differed across reports. In addition, several TAK-003 publications represented overlapping follow-up analyses of the same trial population and were not treated as independent safety datasets. We distinguished between outcomes explicitly reported as absent and outcomes for which no numerical data were provided.

All meta-analyses were performed using Review Manager version 5.4. Forest plots were used to display vaccine-specific estimates for efficacy, hospitalization, and serotype-specific outcomes. The plots included study-level event counts, denominators, absolute event percentages, study weights, risk ratios, and 95% confidence intervals. Prediction intervals were not calculated because no pooled outcome included more than two independent studies; under these conditions, the estimation of between-study variance and the resulting prediction interval would be unstable and potentially misleading.

Because fewer than 10 independent studies were available for any outcome, funnel plots and Egger’s regression were not performed. In addition, several TAK-003 reports represented overlapping follow-up analyses of the same trial population and could not be treated as independent studies. Under these conditions, funnel-plot asymmetry and regression-based tests would have limited statistical power and could produce misleading results. The possibility of publication bias was therefore assessed qualitatively and was considered when interpreting the certainty of the evidence.

### 2.10. Ethical Considerations

This systematic review and meta-analysis were conducted in accordance with the PRISMA 2020 statement and was based exclusively on previously published aggregate data from clinical trial reports and Supplementary Materials. No new participants were recruited, no direct patient contact was performed, and no individual-level identifiable data were collected. Therefore, informed consent was not applicable.

The research protocol was reviewed and approved by the Bioethics Committee of Universidad Privada Antenor Orrego under Resolution No. 01297-2024-UPAO, dated 12 August 2024. The approved project was titled “Eficacia e inmunogenicidad de las vacunas contra el dengue (CYD-TDV y TAK-003) en niños y adolescentes: una revisión sistemática y metaanálisis.” The review protocol was registered in PROSPERO under registration number CRD42024580531. All extracted data were analyzed in aggregate form.

## 3. Results

### 3.1. Study Selection

The database search identified 1462 records from five sources: Cochrane (*n* = 210), Embase (*n* = 82), PubMed (*n* = 133), Scopus (*n* = 828), and Web of Science (*n* = 209). After removal of 404 duplicate records, 1058 unique records were screened by title and abstract. Of these, 1042 records were excluded, and 16 full-text reports were assessed for eligibility. Seven reports were excluded: three because they represented overlapping populations with shorter follow-up or provided no additional relevant outcome information, two because they did not evaluate the target population, and two because they did not meet the inclusion criteria. Overall, nine randomized trial and related placebo-controlled follow-up reports were included in the systematic review (Figure 1; Supplementary Table S1).

### 3.2. Characteristics of Included Randomized Trials and Follow-Up Reports

The included reports evaluated two tetravalent dengue vaccines: CYD-TDV and TAK-003. CYD-TDV was administered as three subcutaneous doses at months 0, 6, and 12, whereas TAK-003 was administered as two subcutaneous doses at months 0 and 3. The reports included children and adolescents aged 2 to 17 years, with follow-up ranging from 9 to 72 months. Four reports evaluated CYD-TDV: one immunogenicity and safety trial conducted in Malaysia, two independent multicentre efficacy trials conducted in Asia and Latin America, and one post hoc long-term follow-up report combining data from the CYD14, CYD15, and CYD23/57 trial programmes. Five reports evaluated TAK-003; however, several TAK-003 reports represented extended follow-up or secondary analyses from the same clinical development programme. Therefore, follow-up reports with overlapping trial populations were not treated as statistically independent in the main meta-analyses (Table 1; Supplementary Table S2).

### 3.3. Risk of Bias

Using RoB 2, most outcome-specific results were judged to have a low risk of bias across the five domains assessed. The immunogenicity and safety results reported by Hss AS et al. (2013) [31] were judged to have some concerns because the blinding of participants and personnel was not described in sufficient detail; for safety, additional concerns remained regarding the potential influence of incomplete blinding on the reporting of subjective adverse events. The long-term hospitalized and severe dengue outcomes reported by Forrat et al. (2021) [15] were also judged to have some concerns because the report was based on post hoc analyses, used several measured or inferred approaches to determine baseline serostatus, and included incomplete long-term follow-up in one of the source trials. The secondary analysis of sequential symptomatic dengue episodes by Sáez et al. (2023) [35] was judged to have some concerns because of uncertainty regarding the selection of the reported result from among multiple potentially eligible analyses. All other assessed results were judged to have a low risk of bias, and no result was classified as having a high risk of bias. Sponsor or manufacturer involvement was documented separately as a contextual funding consideration and was not treated as an additional RoB 2 domain (Supplementary Table S3; Supplementary Figures S1 and S2).

### 3.4. Vaccine Efficacy Against Virologically Confirmed Dengue

For CYD-TDV, the two independent placebo-controlled efficacy trials showed a consistent reduction in virologically confirmed dengue, with no observed statistical heterogeneity (Figure 2; Table 2; Supplementary Figure S3). The absolute risk reduction across these two independent trials was 23.2 fewer cases per 1000 participants (95% CI: 18.9–27.7), corresponding to an NNV of 44 (95% CI: 37–53). For TAK-003, the primary TIDES efficacy report also showed a substantial reduction in virologically confirmed dengue. Within the primary TIDES population, the absolute risk reduction was 23.6 fewer cases per 1000 participants (95% CI: 19.2–28.5), corresponding to an NNV of 43 (95% CI: 36–53). Extended TIDES follow-up reports supported persistence of protection but were summarized descriptively because they included overlapping participants from the same randomized trial population (Figure 2; Table 2; Supplementary Figure S4). Absolute effects were interpreted within each vaccine programme and its corresponding trial context and should not be considered direct comparative measures between CYD-TDV and TAK-003 (Table 3). Detailed study-level efficacy estimates are provided in Supplementary Table S4.

### 3.5. Dengue-Related Hospitalization

Both CYD-TDV and TAK-003 reduced the risk of dengue-related hospitalization compared with placebo (Figure 3; Table 2). For CYD-TDV, the pooled analysis showed moderate heterogeneity, and trial-specific absolute effects differed substantially: the ARR was 12.0 fewer hospitalizations per 1000 participants in the Asian trial (95% CI: 7.5–17.2; NNV = 84, 95% CI: 59–133) and 50.0 fewer per 1000 participants in the Latin American trial (95% CI: 6.2–91.6; NNV = 20, 95% CI: 11–163). These estimates were presented separately because endpoint-specific denominators and baseline hospitalization risks differed substantially between trials (Table 3; Supplementary Figure S7). For TAK-003, the primary TIDES efficacy report showed an absolute reduction of 12.9 dengue-related hospitalizations per 1000 participants (95% CI: 10.2–16.1), corresponding to an NNV of 78 (95% CI: 62–99). Subsequent follow-up reports continued to show protection against hospitalization but were not treated as independent estimates because they represented extended observation of the same randomized TIDES population (Table 3; Supplementary Figure S8). Detailed study-level hospitalization estimates are provided in Supplementary Table S5.

### 3.6. Serotype-Specific Efficacy

Serotype-specific efficacy was presented separately for DENV-1, DENV-2, DENV-3, and DENV-4. No overall pooled estimate across serotypes was used in the main manuscript because combining multiple serotype-specific outcomes from the same participants would create a unit-of-analysis problem.

For CYD-TDV, pooled estimates indicated protection against all four DENV serotypes, with no observed heterogeneity within the serotype-specific analyses. Protection was more pronounced against DENV-3 and DENV-4 than against DENV-1 and DENV-2.

For TAK-003, the primary efficacy report showed lower risks of virologically confirmed dengue for DENV-1, DENV-2, and DENV-3, with the largest relative reduction observed for DENV-2. The DENV-4 estimate was imprecise and should not be interpreted as evidence of a consistent serotype-specific effect. In addition, serotype-specific efficacy varied according to baseline dengue serostatus, particularly during longer follow-up. Therefore, these estimates should be interpreted within the corresponding serostatus and follow-up context rather than as uniform protection across serotypes. Extended follow-up reports were summarized descriptively because they involved overlapping participants from the same clinical trial programme. Detailed serotype-specific estimates are presented in Table 2, Supplementary Table S6, and Supplementary Figures S5 and S6.

### 3.7. Baseline Serostatus and Age-Related Findings

Subgroup data were not sufficiently consistent to support a formal meta-analysis according to baseline serostatus or age. In the primary TAK-003 efficacy report, vaccine efficacy against virologically confirmed dengue was 76.1% (95% CI: 68.5–81.9%) among baseline-seropositive participants and 66.2% (95% CI: 49.1–77.5%) among baseline-seronegative participants. Vaccine efficacy against hospitalized virologically confirmed dengue was 91.4% (95% CI: 81.7–95.9%) and 88.1% (95% CI: 68.5–95.5%) in baseline-seropositive and baseline-seronegative participants, respectively [7]. These subgroup estimates suggested protection in both serostatus groups but did not provide sufficient evidence of a statistically significant interaction between baseline serostatus and vaccine effect.

For CYD-TDV, event counts and denominators stratified simultaneously by baseline serostatus, age, and hospitalization were not reported consistently across the included trial reports. A quantitative comparison of participants aged ≥9 years versus younger participants was also not feasible because age categories differed across vaccine trials and did not consistently correspond to the prespecified 9-year threshold. In addition, aggregate published data did not provide the information required to estimate within-trial interaction effects. Consequently, findings related to baseline serostatus, age, and hospitalization were synthesized narratively and should be interpreted as exploratory.

### 3.8. Severe Dengue

Severe dengue outcomes were summarized narratively because event counts were limited and long-term reports included participants from overlapping CYD-TDV trial populations. In the six-year follow-up analysis by Forrat et al. (2021) [15], CYD-TDV reduced the risk of severe virologically confirmed dengue among baseline-seropositive participants aged ≥9 years, with a hazard ratio of 0.15 (95% CI: 0.06–0.39) using the multiple-imputation method from baseline. Evidence of protection was also observed among baseline-seropositive children aged 6–8 years, although estimates varied according to the method used to determine baseline serostatus.

In contrast, baseline-seronegative recipients showed an unfavorable pattern. Among seronegative participants aged ≥9 years, the estimated risk of severe dengue was higher among vaccine recipients, although the confidence intervals included the null value. Among seronegative participants aged <9 years, an increased risk of hospitalized and severe dengue was observed in the CYD-TDV group. These findings were interpreted as subgroup-specific long-term safety evidence and were not pooled with the primary efficacy trials because the trial populations overlapped.

### 3.9. Immunogenicity

Immunogenicity findings were reported using heterogeneous assays, measurement scales, and summary metrics and were therefore synthesized narratively. For CYD-TDV, the included reports described increases in neutralizing antibody responses against all four DENV serotypes after vaccination, although the magnitude of these responses varied by serotype and follow-up time point.

For TAK-003, neutralizing antibody responses were also observed against the four DENV serotypes. Responses were generally more pronounced for DENV-2 and DENV-1 and differed according to baseline serostatus and follow-up time point. However, study reports used non-equivalent measures, including geometric mean titres, geometric mean ratios, log-transformed titres, seropositivity rates, and PRNT50-based neutralization outcomes. Consequently, no pooled quantitative estimate was calculated, and immunogenicity findings were not directly compared between CYD-TDV and TAK-003. Study-specific findings, including the separate adolescent immunogenicity and safety trial by Biswal et al. (2021) [34], are summarized in Supplementary Table S7.

### 3.10. Certainty of the Evidence

The certainty of the evidence varied across vaccines and outcomes. For virologically confirmed dengue, the certainty was rated as moderate for both CYD-TDV and TAK-003. Certainty for dengue-related hospitalization was low for CYD-TDV because of moderate heterogeneity and differences in endpoint-specific populations and denominators, whereas it was moderate for TAK-003. Evidence for severe dengue was rated as low for both vaccines. For CYD-TDV, certainty was limited by rare events, post hoc analyses, overlapping trial populations, and dependence of the benefit–risk profile on baseline serostatus and age. For TAK-003, certainty was limited by rare events, reliance on one trial programme, and uncertainty for some serotype–serostatus combinations. The certainty of serotype-specific efficacy was moderate for CYD-TDV and low for TAK-003, mainly because of imprecision for some serotypes, particularly DENV-4. Immunogenicity evidence was rated as low for both vaccines because antibody outcomes were heterogeneous and were considered indirect indicators of clinical protection. Safety evidence was rated as low for CYD-TDV and moderate for TAK-003. Detailed GRADE assessments and the reasons for downgrading are presented in Supplementary Table S8.

### 3.11. Safety Outcomes

Safety findings were synthesized narratively because adverse-event definitions, reporting periods, and attribution to vaccination differed across reports. In the CYD-TDV efficacy trials, serious adverse-event frequencies were generally similar between vaccine and placebo recipients. In the Asian trial, serious adverse events occurring within 28 days after vaccination were reported in 54 children in the vaccine group (1%) and 33 children in the placebo group (1%). In the Latin American trial, serious adverse events within 28 days after an injection occurred in 81 vaccine recipients (0.6%) and 40 placebo recipients (0.6%). No consistent short-term imbalance in vaccine-related serious adverse events was identified.

Long-term safety findings differed according to baseline dengue serostatus and age. In the six-year follow-up reported by Forrat et al. (2021) [15], baseline-seropositive participants aged ≥9 years had lower risks of hospitalized and severe virologically confirmed dengue after CYD-TDV vaccination, with hazard ratios of 0.19 (95% CI: 0.12–0.30) and 0.15 (95% CI: 0.06–0.39), respectively. In contrast, an increased risk pattern was observed among baseline-seronegative recipients, particularly those aged <9 years. The attributable excess risk among seronegative participants aged <9 years ranged from 21.8 to 40.0 hospitalized cases and from 7.6 to 11.3 severe cases per 1000 vaccine recipients, depending on the method used to infer baseline serostatus. These findings indicate that the long-term safety profile of CYD-TDV cannot be interpreted independently of previous dengue exposure and age.

For TAK-003, the primary efficacy report documented serious adverse events in 3.1% of vaccine recipients and 3.8% of placebo recipients, without a clinically meaningful imbalance in vaccine-related serious adverse events. During the second year of follow-up, serious adverse events occurred in 271 TAK-003 recipients (2.0%) and 151 placebo recipients (2.3%), and none was considered related to the investigational product or study procedures. Three deaths were reported during this period—two in the TAK-003 group and one in the placebo group—and none were considered vaccine-related. Serious adverse events resulted in withdrawal or study discontinuation in two participants in each group. Long-term follow-up did not identify a new major safety signal or evidence of vaccine-associated enhanced dengue, although safety findings from repeated TAK-003 publications were interpreted as overlapping follow-up from the same clinical trial programme rather than as independent evidence. Detailed safety findings are presented in Supplementary Table S9.

When the long-term safety findings were considered together, the available evidence showed different patterns according to vaccine and baseline serostatus. For CYD-TDV, baseline-seropositive participants aged ≥9 years had lower risks of hospitalized and severe virologically confirmed dengue during six years of follow-up (HR = 0.19, 95% CI: 0.12–0.30, and HR = 0.15, 95% CI: 0.06–0.39, respectively), whereas baseline-seronegative children aged <9 years showed excess risks of hospitalized and severe dengue ranging from 21.8 to 40.0 and from 7.6 to 11.3 cases per 1000 vaccine recipients, respectively. For TAK-003, serious adverse-event frequencies remained similar between vaccine and placebo groups during extended follow-up, and no vaccine-associated enhanced dengue signal was identified; however, these findings were derived largely from repeated follow-up of the same TIDES population rather than from independent long-term safety cohorts. Because follow-up periods, safety definitions, denominators, and subgroup reporting differed across reports, these data were not quantitatively pooled and should be interpreted as a structured long-term safety synthesis rather than as a single summary effect.

### 3.12. Contextual Interpretation of Absolute Effects and Evidence Independence

For CYD-TDV, the combined absolute reduction in virologically confirmed dengue across the two independent efficacy trials was 23.2 cases per 1000 participants, corresponding to an NNV of 44. For dengue-related hospitalization, the absolute effect differed substantially between the two independent CYD-TDV trials—12.0 fewer hospitalizations per 1000 participants in the Asian trial and 50.0 fewer per 1000 participants in the Latin American trial, corresponding to NNVs of 84 and 20, respectively. These trial-specific absolute effects should be interpreted in relation to differences in baseline event risk, endpoint-specific denominators, population characteristics, and epidemiological setting rather than as a uniform estimate of clinical benefit across settings.

For TAK-003, the primary TIDES efficacy report showed an absolute reduction of 23.6 virologically confirmed dengue cases per 1000 participants, corresponding to an NNV of 43, and an absolute reduction of 12.9 dengue-related hospitalizations per 1000 participants, corresponding to an NNV of 78. These estimates describe absolute benefit within the randomized TIDES population and are not intended for statistical or clinical comparison with the CYD-TDV estimates.

Subsequent TAK-003 publications provided progressively longer follow-up of the randomized TIDES population and added information on durability of protection, hospitalization, serotype-specific performance, immunogenicity, and long-term safety. Because these reports involved overlapping participants from the same trial programme, they were not considered additional independent efficacy replications. This distinction concerns the structure and independence of the evidence rather than the validity or magnitude of the vaccine effects reported during extended follow-up.

### 3.13. Summary of Evidence and Heterogeneity

The magnitude and certainty of the observed effects varied across vaccine programmes and outcomes. The moderate heterogeneity in the CYD-TDV hospitalization analysis was consistent with differences in participant age, geographic setting, baseline dengue risk, endpoint-specific denominators, surveillance procedures, and follow-up. For TAK-003, heterogeneity across independent studies could not be formally assessed because the principal efficacy evidence was derived from one trial programme, with subsequent TIDES publications representing overlapping follow-up analyses. These differences limit cross-programme comparisons and support interpreting each vaccine within its own clinical and epidemiological context rather than inferring comparative superiority.

## 4. Discussion

The present systematic review and meta-analysis confirm the previously reported protective effects of CYD-TDV and TAK-003 against virologically confirmed dengue and dengue-related hospitalization in children and adolescents. The principal contribution of the present synthesis concerns how the strength and structure of this evidence are interpreted when trial independence, baseline risk, absolute effects, and follow-up duration are considered together. For CYD-TDV, the two independent efficacy trials supported reductions in virologically confirmed dengue and hospitalization, although the absolute hospitalization benefit varied substantially between trial populations. For TAK-003, the primary TIDES trial supported reductions in virologically confirmed dengue and hospitalization, while subsequent publications extended follow-up of the same randomized population rather than providing additional independent efficacy replications. These findings are interpreted separately within each vaccine programme and provide the framework for the discussion of efficacy, heterogeneity, serotype-specific effects, baseline serostatus, immunogenicity, and long-term safety that follows.

Previous systematic reviews have already characterized the efficacy, immunogenicity, and safety of dengue vaccines. Godói et al. evaluated the efficacy, immunogenicity, and safety of CYD-TDV, while Rosa et al. similarly synthesized these outcomes in children and adolescents. Flacco et al. focused on TAK-003, including efficacy according to baseline serostatus, whereas Pizzol et al. provided a broader synthesis across dengue vaccines, populations, and study designs [12,16,19,21]. These studies establish that the principal efficacy and safety profiles of CYD-TDV and TAK-003 are not newly identified by the present review. Accordingly, our contribution should be interpreted as an incremental synthesis of the existing placebo-controlled evidence rather than as the identification of a previously unreported vaccine effect.

The present analysis differs primarily in how the structure of the available evidence was handled and interpreted. Successive publications were linked to their source randomized trial populations, allowing extended follow-up to be distinguished from independent replication and preventing overlapping populations from being treated as independent evidence. Relative and absolute effects were interpreted within each vaccine programme and its corresponding baseline risk rather than as direct comparisons between CYD-TDV and TAK-003. This approach is particularly relevant for TAK-003, for which several publications represent progressively longer follow-up of the TIDES population, and for CYD-TDV, for which two independent efficacy trials contributed to the principal pooled estimates. Absolute risk reductions and numbers needed to vaccinate were therefore used as context-dependent measures of clinical impact, not as evidence of comparative superiority between vaccines. Taken together, these analyses refine the interpretation of the strength, independence, and clinical applicability of the existing evidence while acknowledging that efficacy, immunogenicity, and safety have already been extensively addressed in previous reviews.

First, our findings indicated that both vaccines reduced the risk of virologically confirmed dengue within their respective placebo-controlled trial programmes. For CYD-TDV, the observed RR of 0.41, corresponding to an approximate 59% relative risk reduction, was consistent with the pivotal CYD14 and CYD15 programmes, which reported vaccine efficacies of 56.5% in Asia and 60.8% in Latin America, respectively, as well as 59.2% in the pooled per-protocol analysis [32,33]. This finding was also consistent with the meta-analysis by Rosa et al. [19], which reported an overall efficacy of approximately 60% and an RR of 0.40. For TAK-003, the observed RR of 0.28 indicated a substantial reduction within the TIDES trial programme. The primary analysis reported an efficacy of 80.2% [36], whereas the subsequent 18-month analysis reported an efficacy of 73.3% [7]. Nevertheless, the effect estimates for CYD-TDV and TAK-003 should not be compared directly because the respective trials differed in design, participant characteristics, baseline serostatus, geographic and epidemiological settings, circulating serotypes, outcome definitions, and duration of follow-up. Moreover, no head-to-head trial or formal network meta-analysis were conducted.

Second, dengue-related hospitalization was reduced within each vaccine programme. For CYD-TDV, the pooled estimate from the two independent efficacy trials showed a reduced risk of hospitalization (RR = 0.41; 95% CI: 0.25–0.67). This finding was consistent with the CYD15 trial, in which CYD-TDV reduced dengue-related hospitalization by 80.3%, and with long-term analyses showing sustained protection against hospitalized and severe virologically confirmed dengue among baseline-seropositive participants, particularly those aged 9 years or older [15,33,37]. In Forrat et al. (2021) [15], CYD-TDV showed strong protection over six years in baseline-seropositive participants aged ≥9 years, with hazard ratios of 0.19 for hospitalized VCD and 0.15 for severe VCD. For TAK-003, the hospitalization finding was also consistent with the TIDES programme, which reported 90.4% efficacy against hospitalized VCD at 18 months, 83.6% at three years, and persistent protection during longer-term follow-up [7,9,10]. Hospitalization is a particularly relevant clinical and public health endpoint because it reflects the potential of vaccination to reduce severe disease and pressure on health systems during dengue outbreaks.

The moderate heterogeneity observed in the CYD-TDV hospitalization analysis (I^2^ = 56%) was plausibly related to important clinical and methodological differences between the two contributing trials. The Asian trial enrolled children aged 2–14 years, whereas the Latin American trial included participants aged 9–16 years, resulting in different age distributions and likely different baseline dengue immunity profiles. The trials were also conducted in distinct epidemiological settings with differences in dengue transmission intensity, circulating serotypes, healthcare access, surveillance procedures, hospitalization practices, and follow-up. Importantly, the endpoint-specific denominators and baseline hospitalization risks differed substantially between studies, which was also reflected in the absolute effects: the ARR was 12.0 per 1000 participants in the Asian trial and 50.0 per 1000 participants in the Latin American trial. These differences are therefore plausible contributors to the observed between-study heterogeneity. However, because only two independent trials contributed to the pooled estimate, formal subgroup analysis, meta-regression, or leave-one-out sensitivity analysis could not provide a reliable assessment of the source of heterogeneity. Accordingly, no single factor can be identified as the definitive explanation for the observed I^2^.

Third, our serotype-specific findings supported the concept that dengue vaccine performance was not uniform across DENV serotypes. CYD-TDV showed protection against DENV-1, DENV-2, DENV-3, and DENV-4, which was consistent with evidence from the CYD14 and CYD15 trials showing stronger protection against DENV-3 and DENV-4 than against DENV-1 and DENV-2. In CYD15, serotype-specific efficacy was 50.3% for DENV-1, 42.3% for DENV-2, 74.0% for DENV-3, and 77.7% for DENV-4 [32,33]. For TAK-003, the primary TIDES analysis showed lower risks of virologically confirmed dengue for DENV-1, DENV-2, and DENV-3, with the largest relative reduction observed for DENV-2, whereas the DENV-4 estimate remained imprecise [7,36]. During longer follow-up, serotype-specific efficacy also differed according to baseline dengue serostatus. Among baseline-seropositive participants, protection was observed across all four serotypes, whereas among baseline-seronegative participants, the most consistent evidence was for DENV-1 and DENV-2, with greater uncertainty for DENV-3 and DENV-4 [9]. These findings indicate that TAK-003 serotype-specific effects should be interpreted according to baseline serostatus, follow-up duration, and circulating serotype rather than as uniform protection across all DENV serotypes.

Fourth, baseline dengue serostatus is essential for interpreting vaccine efficacy and safety, particularly for CYD-TDV. Within the evidence included in this review, the six-year follow-up analysis by Forrat et al. [15] showed sustained protection against hospitalized and severe virologically confirmed dengue among baseline-seropositive participants, particularly those aged ≥9 years. In contrast, baseline-seronegative recipients showed an unfavorable long-term risk pattern, especially among younger children. These findings were consistent with the external individual-level reanalysis by Sridhar et al. [37], which reported protection among seropositive recipients but an increased risk of hospitalized and severe dengue among seronegative vaccine recipients. For TAK-003, baseline serostatus was also associated with differences in vaccine performance. The 18-month analysis showed efficacy of 76.1% among baseline-seropositive participants and 66.2% among baseline-seronegative participants, while the three-year analysis reported corresponding estimates of 65.0% and 54.3% [7,9]. Nevertheless, efficacy among baseline-seronegative participants was mainly driven by protection against DENV-1 and DENV-2, with remaining uncertainty for DENV-3 and DENV-4. Therefore, baseline serostatus remains central to both individual risk assessment and population-level vaccination policy.

However, the available aggregate data did not permit formal within-trial comparisons between baseline-seropositive and baseline-seronegative participants, children aged ≥9 years and those aged <9 years, or the interaction between baseline serostatus and hospitalization risk. Differences across these subgroups should therefore be interpreted as descriptive patterns rather than as statistically confirmed effect modification. This limitation is particularly relevant for CYD-TDV, for which age and previous dengue exposure jointly influence the benefit–risk profile, and for TAK-003, for which protection among baseline-seronegative participants varied across serotypes.

Fifth, safety was evaluated separately from the absolute efficacy measures described above. The ARR and NNV estimates quantify absolute benefit for clinical efficacy outcomes and were not used to infer vaccine safety. Safety was assessed using serious adverse events, vaccine-related serious adverse events, deaths, withdrawals or study discontinuations due to adverse events, and clinically relevant hospitalized or severe dengue outcomes during longer-term follow-up, including analyses according to baseline serostatus when available. In the pivotal trials, serious adverse-event frequencies were generally similar between vaccine and placebo recipients for both CYD-TDV and TAK-003, and no consistent imbalance in events attributed directly to vaccination was identified [7,32,33]. These findings support acceptable short-term tolerability. However, conventional adverse-event summaries do not fully capture clinically relevant vaccine safety in dengue, because hospitalized or severe dengue occurring after vaccination may emerge during subsequent natural infection rather than immediately after vaccine administration.

This distinction is particularly important for CYD-TDV. The included six-year follow-up analysis showed that long-term hospitalized and severe dengue outcomes differed substantially according to baseline serostatus and age [15]. These findings were consistent with the external reanalysis by Sridhar et al. [37], which also identified an increased risk of hospitalized and severe dengue among baseline-seronegative recipients. Consequently, the safety of CYD-TDV cannot be interpreted independently of reliable evidence of previous dengue exposure. For TAK-003, the available trial programme did not identify a comparable enhanced-disease signal during long-term follow-up, and serious adverse events, deaths, and study discontinuations were generally balanced between groups [7,8,9,10]. Nevertheless, continued surveillance remains warranted, particularly among baseline-seronegative recipients and for serotypes with less certain efficacy.

The biological mechanism underlying this safety signal has not been established definitively, but the leading explanation is that CYD-TDV may immunologically prime a dengue-seronegative recipient in a manner resembling a silent primary DENV infection. When the vaccinated individual subsequently experiences a first natural dengue infection, the immune response may resemble that observed during a secondary infection, which is associated with a higher risk of severe disease. Cross-reactive antibodies at subneutralizing concentrations may facilitate viral entry into Fcγ receptor-bearing cells through antibody-dependent enhancement, potentially increasing viral replication and downstream inflammatory responses. Other vaccine-specific factors, including the absence of DENV nonstructural proteins in the yellow fever virus backbone, may also contribute to differences in cellular and humoral immunity; however, their precise contribution remains uncertain [37,38,39].

Recognition of this risk led to a major change in the recommended use of CYD-TDV. Vaccination was restricted to individuals with reliable evidence of previous dengue infection, and pre-vaccination screening became necessary to minimize the possibility of vaccinating seronegative recipients. Thus, the CYD-TDV experience demonstrated that overall trial efficacy and conventional short-term adverse-event profiles are insufficient to establish dengue vaccine safety without considering baseline serostatus and delayed outcomes occurring after natural DENV exposure [39,40].

Sixth, the immunogenicity findings provided biological plausibility for the observed clinical protection, but they should not be treated as direct substitutes for efficacy outcomes. CYD-TDV increased neutralizing antibody titers against the four dengue serotypes, and Rosa et al. [19] reported positive immunogenicity estimates for DENV-1, DENV-2, DENV-3, and DENV-4. These findings supported the capacity of CYD-TDV to induce tetravalent humoral responses, although clinical efficacy still varied by serotype and baseline serostatus. For TAK-003, the finding of more pronounced responses against DENV-2 and DENV-1 was consistent with mechanistic immunogenicity studies showing that TAK-003 induces particularly high levels of DENV-2 type-specific neutralizing antibodies, whereas responses against DENV-1, DENV-3, and DENV-4 may contain a larger cross-reactive component [41]. DeMaso et al. [42] also reported broad tetravalent type-specific and cross-reactive neutralizing antibody responses, while Sirivichayakul et al. [43] showed persistence of neutralizing antibody titers up to 36 months. Thus, the immunogenicity data were supportive, but the absence of a universally accepted correlate of protection against DENV infection means that neutralizing antibody titres should be interpreted as complementary evidence rather than as definitive proof of clinical efficacy.

Finally, the heterogeneity observed across vaccines and outcomes was biologically and methodologically plausible. CYD-TDV and TAK-003 differed in vaccine platform, dosing schedule, antigenic backbone, age range, baseline serostatus distribution, countries included, follow-up duration, and circulating serotypes. CYD-TDV was evaluated in large Asian and Latin American trials using a three-dose schedule and showed efficacy that was strongly influenced by age and baseline serostatus [32,33,37,44]. TAK-003 was evaluated using a two-dose schedule in children and adolescents from eight dengue-endemic countries, with efficacy that declined over time for symptomatic VCD but remained more durable for hospitalized VCD [7,9,10]. Differences in hospitalization criteria, intensity of surveillance, dominant serotypes, and local transmission dynamics may also explain variation across reports. Therefore, the present findings should be interpreted as a product-specific synthesis rather than as evidence of direct vaccine ranking. From a public health perspective, absolute effects should be interpreted within each vaccine programme because they depend on baseline event risk, population characteristics, follow-up duration, and epidemiological context. In addition, a larger number of follow-up publications should not be interpreted as a larger number of independent trial populations. Clinical and policy interpretation should therefore consider product-specific absolute benefit, baseline risk, evidence independence, baseline serostatus, serotype epidemiology, and the local implementation context.

These findings should be interpreted in light of current World Health Organization recommendations. WHO recommends TAK-003 for children aged 6–16 years living in settings with high dengue transmission intensity, using a two-dose schedule administered at least 3 months apart. Programmatic use is not currently recommended in low- or moderate-transmission settings because the efficacy–risk profile against DENV-3 and DENV-4 among baseline-seronegative individuals remains insufficiently characterized. WHO also recommends that countries prioritize geographical areas in which dengue represents a substantial public health problem and consider targeted subnational introduction when transmission intensity varies within a country [40,45].

The 2024 WHO position paper described the previous product-specific recommendation that CYD-TDV should be administered only to individuals with evidence of prior dengue infection. However, updated WHO information published in April 2025 indicates that TAK-003 is currently the only dengue vaccine available for prevention [40,45]. Consequently, vaccine policy should reflect current product availability and should not be based solely on the magnitude of relative efficacy estimates. National immunization programmes should consider age-specific hospitalization patterns, local seroprevalence, circulating serotypes, geographical variation in transmission intensity, and the capacity to complete the recommended vaccination schedule. Dengue vaccination should remain part of an integrated control strategy that includes vector control, effective case management, epidemiological surveillance, community education, and community engagement [45,46].

Several evidence gaps remain relevant to policy and implementation when the included trial evidence is considered together with complementary external analyses. First, no head-to-head randomized trial has directly compared CYD-TDV with TAK-003, and differences between trial programmes prevent reliable vaccine ranking based on numerical efficacy estimates alone. Second, individual participant data analyses are needed to evaluate interactions among baseline serostatus, age, infecting serotype, transmission intensity, and the risk of hospitalized or severe dengue using consistent definitions. Third, additional evidence is required on the durability of protection, the potential need and optimal timing of booster doses, and long-term safety among baseline-seronegative recipients, particularly for serotypes with less certain protection. Real-world studies should also assess vaccine effectiveness during outbreaks, rare adverse outcomes, completion of multidose schedules, and performance across populations that were underrepresented in the clinical trials. Finally, implementation research should examine the accuracy, feasibility, and cost-effectiveness of pre-vaccination screening for CYD-TDV, as well as the epidemiological and operational criteria required for geographically targeted TAK-003 programmes. Robust post-introduction surveillance and pharmacovigilance will therefore be essential to determine whether trial-based benefits are maintained under routine conditions [9,10,15,37,40].

To place the present synthesis more fully within the existing literature, we also considered additional publications identified during peer review. Malisheni et al. previously synthesized the efficacy, safety, and immunogenicity of CYD-TDV in children [18], while Okoye et al. evaluated efficacy, immune response, and safety across dengue vaccine trials in pediatric populations [23]. Bengolea et al. specifically assessed TAK-003 using randomized placebo-controlled trials, RoB 2, GRADE, and both relative and absolute measures of effect [20], and Naderian et al. reviewed the efficacy, immune response, and safety of both CYD-TDV and TAK-003 in adolescents [24]. Broader recent publications by Wang et al., Kant et al., and Wilder-Smith et al. further addressed the efficacy, safety, limitations, development, or programmatic deployment of current dengue vaccines [25,26,27]. In addition, Gianguzzo et al. provided complementary post-marketing pharmacovigilance evidence for CYD-TDV and TAK-003, although this study was not a systematic review and was outside the placebo-controlled eligibility criteria of the present synthesis [47]. Considered together, this literature confirms that efficacy, immunogenicity, safety, and even absolute-effect estimation have already been addressed in previous publications. Our contribution is therefore positioned more narrowly around the explicit identification of independent trial populations, the handling of overlapping follow-up reports, and the interpretation of relative and absolute effects within the corresponding vaccine programme and epidemiological context (Supplementary Table S10).

The contribution of the present synthesis concerns the organization and interpretation of the existing trial evidence rather than the identification of a previously unreported vaccine effect. For CYD-TDV, absolute effects were estimated from two independent efficacy trials, with substantial variation in trial-specific hospitalization benefit that underscores the influence of baseline risk and study context. For TAK-003, the primary TIDES report provided the principal efficacy and absolute-effect estimates, whereas subsequent publications extended follow-up of the same randomized population. By explicitly linking publications to their source trial populations, the present review distinguishes extended observation from independent replication and avoids treating overlapping reports as separate efficacy evidence. Absolute effects are therefore interpreted within each vaccine programme rather than across vaccines. These features do not alter the established efficacy or safety profiles of CYD-TDV and TAK-003; instead, they provide a more transparent assessment of the independence, applicability, and certainty of the available evidence.

This review also has several methodological strengths that support the interpretation of the synthesis-level observations described above. Primary trials and overlapping follow-up reports were identified explicitly, reducing the risk of double counting participants while preserving relevant long-term information. Relative effect estimates were complemented with baseline risks, absolute risk reductions, and numbers needed to vaccinate to provide context-specific measures of clinical impact; however, the use of absolute effects is not presented as a novel methodological contribution because similar measures have been reported in previous evidence syntheses. Certainty was assessed separately for each vaccine and outcome using RoB 2 and GRADE, and long-term hospitalized and severe dengue outcomes were incorporated into the safety assessment according to baseline serostatus when reported. The principal distinguishing methodological feature of the present review is the explicit mapping of successive publications to their source randomized trial populations, which allowed extended follow-up to be separated from independent replication and reduced the risk of double counting overlapping populations. These features strengthen the transparency and interpretability of the synthesis without implying the identification of a new vaccine effect.

An important limitation is that CYD-TDV and TAK-003 were not evaluated in head-to-head trials. Any cross-vaccine interpretation is therefore indirect and highly uncertain because the two clinical trial programmes differed in study design, participant age distribution, baseline dengue seroprevalence, geographic and epidemiological settings, follow-up duration, circulating serotypes, and endpoint definitions. Accordingly, numerical differences in RR, ARR, or NNV should not be interpreted as comparative efficacy between the two vaccines.

Another important limitation is the incomplete reporting of long-term safety outcomes for clinically relevant subgroups. Stratified event counts and denominators according to baseline serostatus and age were not reported consistently across the included trials, and several long-term analyses were derived from overlapping trial populations. Consequently, these outcomes could not be pooled reliably or examined in greater depth, and subgroup-specific conclusions, particularly for baseline-seronegative recipients and younger children, should be interpreted cautiously. The analysis also relied on published aggregate rather than individual participant data, which limited our ability to standardize subgroup definitions, apply a consistent 9-year age threshold, and formally evaluate interactions among baseline serostatus, age, and hospitalization risk.

External validity is also limited because the included trials were conducted within specific epidemiological and programmatic contexts. Vaccine performance observed in these trial populations may not fully generalize to settings with different dengue transmission intensity, baseline seroprevalence, dominant circulating serotypes, healthcare access, or vaccine implementation conditions. Phase IV and other post-implementation effectiveness studies are therefore needed to determine whether the trial-based efficacy and safety profiles are maintained under routine vaccination conditions and across changing epidemiological settings. Publication bias also remains a potential limitation and was considered qualitatively in the GRADE assessment. Finally, because no formal network meta-analysis was conducted, numerical differences between vaccines should be interpreted descriptively rather than as evidence of comparative superiority.

## 5. Conclusions

CYD-TDV and TAK-003 reduced virologically confirmed dengue and dengue-related hospitalization within their respective placebo-controlled trial programmes. For CYD-TDV, the principal efficacy evidence was derived from two independent randomized trials, and the substantial variation in trial-specific absolute hospitalization effects highlights the influence of baseline event risk, population characteristics, and epidemiological context. For TAK-003, the principal efficacy evidence was derived from the TIDES programme, while subsequent publications extended follow-up of the same randomized population rather than providing additional independent efficacy replications.

The findings should therefore be interpreted at the level of each vaccine programme rather than as a direct comparison between CYD-TDV and TAK-003. Absolute effects provide context-specific information on clinical benefit but should be considered alongside relative efficacy, baseline risk, follow-up duration, serotype distribution, and baseline serostatus. Safety also requires product-specific interpretation, particularly because the long-term benefit–risk profile of CYD-TDV depends strongly on previous dengue exposure and age, whereas the available TAK-003 trial programme has not identified a comparable enhanced-disease signal during extended follow-up. Overall, distinguishing independent trial populations from overlapping follow-up reports provides a clearer assessment of the strength, applicability, and certainty of the existing dengue vaccine evidence.

## Figures and Tables

**Figure 1:**
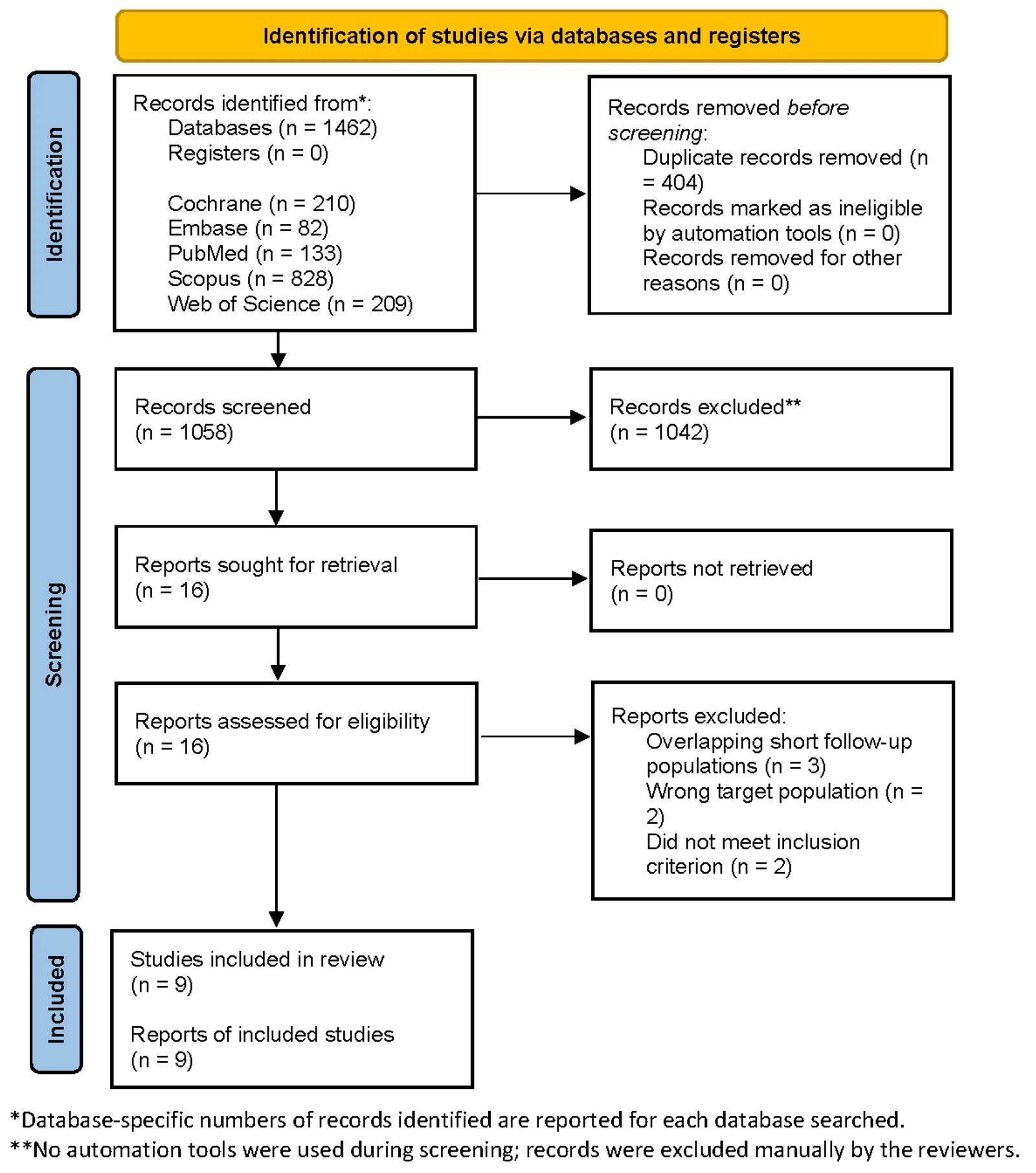
PRISMA 2020 flow diagram of study selection. The search identified 1462 records from Cochrane, Embase, PubMed, Scopus, and Web of Science. After removing 404 duplicate records, 1058 records were screened by title and abstract. Sixteen full-text reports were assessed for eligibility, and nine randomized trial and related placebo-controlled follow-up reports were included in the systematic review.

**Figure 2:**
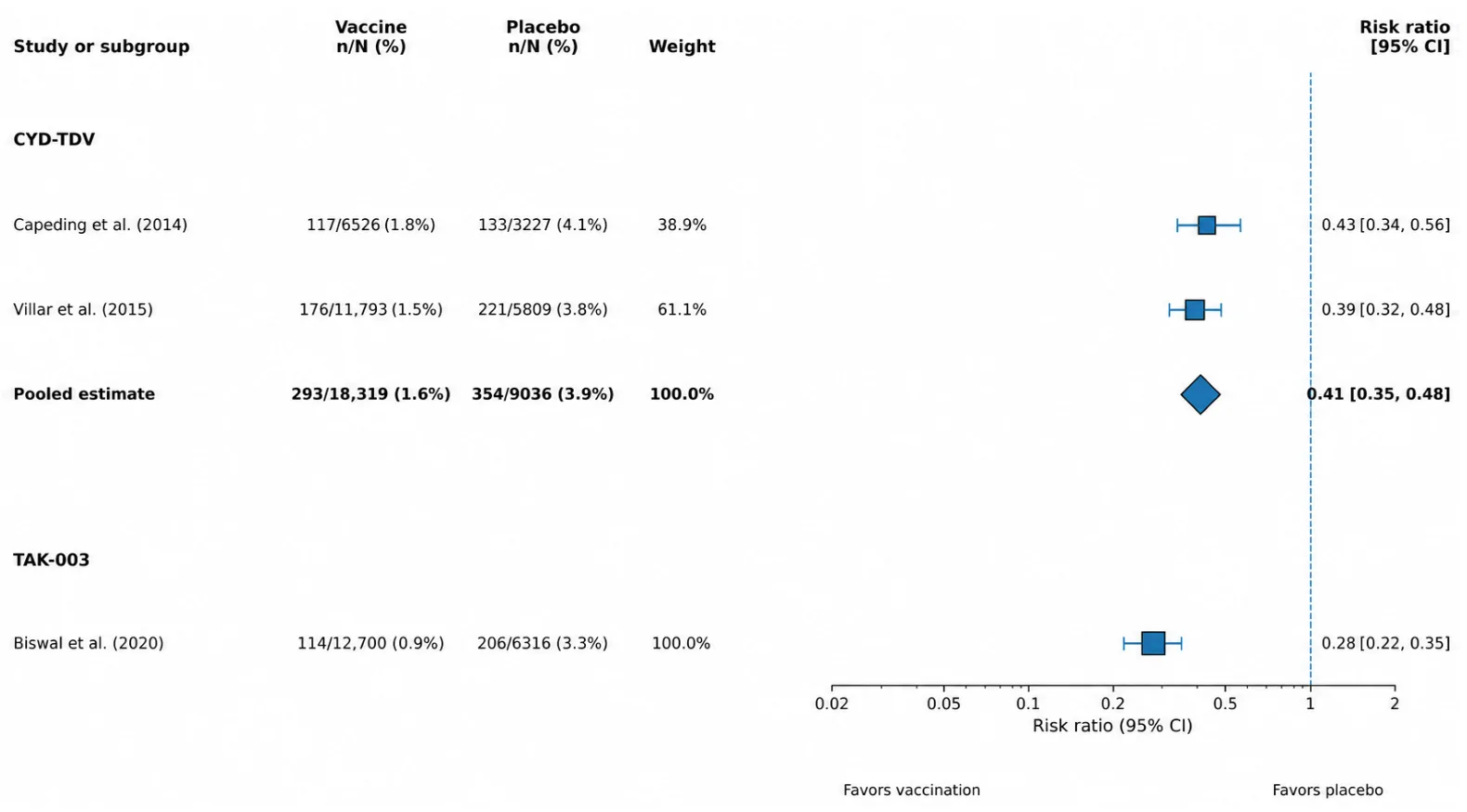
Placebo-controlled efficacy against virologically confirmed dengue, presented separately by vaccine. The CYD-TDV panel includes the two independent randomized efficacy trials by Capeding et al. [32] and Villar et al. [33] and their pooled estimate. The TAK-003 panel displays the primary efficacy report by Biswal et al. [7] only to avoid double counting participants from overlapping follow-up publications. Event counts, denominators, absolute event percentages, study weights, risk ratios, and 95% confidence intervals are shown. Prediction intervals were not calculated because the pooled CYD-TDV analysis included only two independent studies, while the TAK-003 panel was based on a single independent trial. Risk ratios below 1 favor vaccination. Blue squares represent the study-specific effect estimates, with their size proportional to study weight; horizontal lines represent the corresponding 95% CIs. The diamond represents the pooled effect estimate for CYD-TDV.

**Figure 3:**
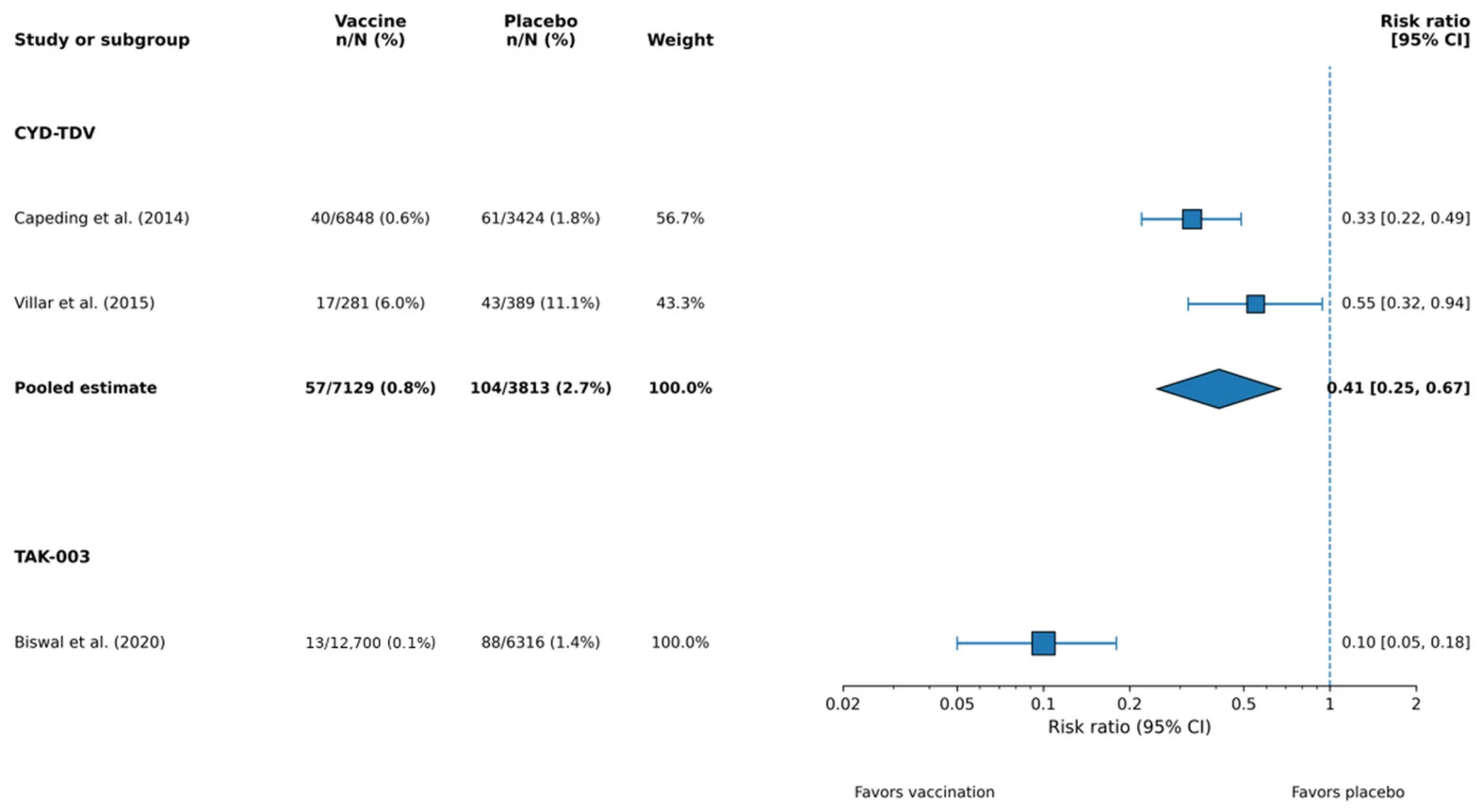
Dengue-related hospitalization risk, presented separately by vaccine. The CYD-TDV panel includes the two independent randomized efficacy trials by Capeding et al. [32] and Villar et al. [33] and their pooled estimate. The TAK-003 panel displays the primary efficacy report by Biswal et al. [7] only to avoid double counting participants from overlapping follow-up publications. Event counts, denominators, absolute event percentages, study weights, risk ratios, and 95% confidence intervals are shown. Hospitalization denominators correspond to the populations or periods at risk reported for this endpoint and may differ from those used for virologically confirmed dengue. Prediction intervals were not calculated because the CYD-TDV analysis included only two independent studies and the TAK-003 panel was based on a single independent trial. Risk ratios below 1 favor vaccination. Blue squares represent the study-specific effect estimates, with their size proportional to study weight; horizontal lines represent the corresponding 95% CIs. The diamond represents the pooled effect estimate for CYD-TDV.

**Table 1:** Characteristics and analytic role of the included dengue vaccine trial reports.

Report	Vaccine/Trial Programme	Setting	Participants, *n*	Age	Schedule	Follow-Up	Main Outcomes	Analytic Role
**Hss AS et al. (2013) [31]**	CYD-TDV immunogenicity/safety trial	Malaysia	250	2–11 years	3 doses: months 0, 6, 12	18 months	Immunogenicity; safety	Narrative immunogenicity and safety synthesis
**Capeding et al. (2014) [32]**	CYD-TDV; CYD14	Multicentre, Asia	10,275	2–14 years	3 doses: months 0, 6, 12	25 months	VCD; hospitalization; severe dengue; serotype-specific efficacy; immunogenicity; safety	Main CYD-TDV efficacy and hospitalization meta-analysis
**Villar et al. (2015) [33]**	CYD-TDV; CYD15	Multicentre, Latin America	20,869	9–16 years	3 doses: months 0, 6, 12	25 months	VCD; hospitalization; severe dengue; serotype-specific efficacy; immunogenicity; safety	Main CYD-TDV efficacy and hospitalization meta-analysis
**Forrat et al. (2021) [15]**	CYD-TDV; six-year follow-up of CYD14, CYD15, and CYD23/57	Asia, Latin America, and Thailand	35,146 across source trials	2–16 years	3 doses: months 0, 6, 12	72 months	Hospitalized VCD; severe VCD; long-term safety by serostatus and age	Long-term subgroup and safety synthesis; not treated as independent pooled evidence
**Biswal et al. (2020) [7]**	TAK-003; TIDES pivotal phase 3 trial	Eight dengue-endemic countries	20,099	4–16 years	2 doses: months 0, 3	21 months	VCD; hospitalization; severe dengue; serotype-specific efficacy; immunogenicity; safety	Main TAK-003 efficacy, hospitalization, and absolute-effect estimates
**Biswal et al. (2021) [34]**	TAK-003 adolescent immunogenicity/safety trial	Mexico	400	12–17 years	2 doses: months 0, 3	9 months	Immunogenicity; safety	Narrative immunogenicity and safety synthesis
**Lopez et al. (2022) [8]**	TAK-003; TIDES extended follow-up	Eight dengue-endemic countries	20,099	4–16 years	2 doses: months 0, 3	27 months	VCD; hospitalization; severe dengue; serotype-specific efficacy; immunogenicity; safety	Descriptive follow-up; overlapping TIDES population
**Sáez et al. (2023) [35]**	TAK-003; TIDES secondary/follow-up analysis	Eight dengue-endemic countries	20,063	4–16 years	2 doses: months 0, 3	Up to 57 months	Sequential symptomatic dengue; hospitalization	Secondary analysis; overlapping TIDES population
**Tricou et al. (2024) [10]**	TAK-003; TIDES long-term follow-up	Eight dengue-endemic countries	20,099	4–16 years	2 doses: months 0, 3	57 months	VCD; hospitalization; severe dengue; serotype-specific efficacy; immunogenicity; long-term safety	Long-term descriptive synthesis; overlapping TIDES population

Abbreviations: CYD-TDV, chimeric yellow fever–dengue tetravalent vaccine; TAK-003, tetravalent dengue vaccine candidate; TIDES, Tetravalent Immunization against Dengue Efficacy Study; VCD, virologically confirmed dengue. All included efficacy trials used saline-based placebo comparators. Capeding et al. (2014) [32] and Villar et al. (2015) [33] represented independent CYD-TDV efficacy trials. Forrat et al. (2021) [15] included overlapping participants from CYD14 and CYD15 and was therefore retained for long-term subgroup and safety analyses but not treated as statistically independent evidence. Lopez et al. (2022) [8], Sáez et al. (2023) [35], and Tricou et al. (2024) [10] represented overlapping follow-up analyses of the TIDES programme and were summarized descriptively rather than pooled as independent studies.

**Table 2:** Main efficacy and serotype-specific estimates by dengue vaccine.

Outcome	Vaccine	Evidence Base	Effect Estimate	Heterogeneity/Interpretation
Virologically confirmed dengue	CYD-TDV	Capeding et al. (2014) [32] and Villar et al. (2015) [33]; two independent trials	RR 0.41 (95% CI: 0.35–0.48)	I^2^ = 0%
Virologically confirmed dengue	TAK-003	Biswal et al. (2020) [7]; primary TIDES efficacy report	RR 0.28 (95% CI: 0.22–0.35)	Not applicable; one principal trial programme
Dengue-related hospitalization	CYD-TDV	Capeding et al. (2014) [32] and Villar et al. (2015) [33]; two independent trials	RR 0.41 (95% CI: 0.25–0.67)	I^2^ = 56%; moderate heterogeneity
Dengue-related hospitalization	TAK-003	Biswal et al. (2020) [7]; primary TIDES efficacy report	RR 0.10 (95% CI: 0.05–0.18)	Not applicable; one principal trial programme
DENV-1	CYD-TDV	Two independent efficacy trials	RR 0.50 (95% CI: 0.39–0.64)	I^2^ = 0%
DENV-2	CYD-TDV	Two independent efficacy trials	RR 0.60 (95% CI: 0.45–0.81)	I^2^ = 0%
DENV-3	CYD-TDV	Two independent efficacy trials	RR 0.25 (95% CI: 0.18–0.35)	I^2^ = 0%
DENV-4	CYD-TDV	Two independent efficacy trials	RR 0.23 (95% CI: 0.16–0.35)	I^2^ = 0%
DENV-1	TAK-003	Biswal et al. (2020) [7]; primary TIDES efficacy report	RR 0.30 (95% CI: 0.20–0.46)	Single programme estimate
DENV-2	TAK-003	Biswal et al. (2020) [7]; primary TIDES efficacy report	RR 0.05 (95% CI: 0.02–0.10)	Single programme estimate
DENV-3	TAK-003	Biswal et al. (2020) [7]; primary TIDES efficacy report	RR 0.52 (95% CI: 0.37–0.74)	Single programme estimate
DENV-4	TAK-003	Biswal et al. (2020) [7]; primary TIDES efficacy report	RR 0.50 (95% CI: 0.14–1.72)	Imprecise estimate

Abbreviations: CI, confidence interval; DENV, dengue virus; RR, risk ratio; TIDES, Tetravalent Immunization against Dengue Efficacy Study. CYD-TDV estimates were pooled from two independent efficacy trials. For TAK-003, the primary TIDES efficacy report was used for the main estimates because later publications involved overlapping participants and were not treated as independent evidence. Serotype-specific outcomes were analyzed separately; no overall cross-serotype estimate was calculated because these outcomes were correlated within the same trial populations.

**Table 3:** Absolute effects of vaccination on virologically confirmed dengue and dengue-related hospitalization.

Outcome	Vaccine/Evidence Base	Vaccine Events/Total	Placebo Events/Total	Vaccine Risk per 1000	Baseline Placebo Risk per 1000	Absolute Risk Reduction per 1000 (95% CI)	Number Needed to Vaccinate (95% CI)
Virologically confirmed dengue	CYD-TDV, two independent trials combined	293/18,319	354/9036	16.0	39.2	23.2 (18.9–27.7)	44 (37–53)
Virologically confirmed dengue	TAK-003, primary efficacy report	114/12,700	206/6316	9.0	32.6	23.6 (19.2–28.5)	43 (36–53)
Dengue-related hospitalization	CYD-TDV, Capeding et al. (2014) [32]	40/6848	61/3424	5.8	17.8	12.0 (7.5–17.2)	84 (59–133)
Dengue-related hospitalization	CYD-TDV, Villar et al. (2015) [33]	17/281	43/389	60.5	110.5	50.0 (6.2–91.6)	20 (11–163)
Dengue-related hospitalization	TAK-003, primary efficacy report	13/12,700	88/6316	1.0	13.9	12.9 (10.2–16.1)	78 (62–99)

Note: Baseline risk was defined as the observed event proportion in the placebo group. Absolute risk reduction (ARR) was calculated as the placebo-group risk minus the vaccine-group risk and is expressed as fewer events per 1000 participants. Ninety-five percent confidence intervals for ARR were calculated using the Newcombe method based on Wilson score intervals for two independent proportions. The number needed to vaccinate (NNV) was calculated as the reciprocal of the ARR; its 95% confidence interval was obtained by inverting the corresponding ARR confidence limits. NNV point estimates and confidence limits were rounded up to the nearest whole number. Absolute effects depend on baseline risk, follow-up duration, population characteristics, and endpoint definition and should not be interpreted as direct comparative measures between vaccines. CYD-TDV hospitalization estimates were presented separately because trial-specific denominators and baseline risks differed substantially.

## Data Availability

The data analyzed in this study were extracted from published clinical trial reports and their Supplementary Materials. The extraction sheet, analytic dataset, and Review Manager files are available from the corresponding author upon reasonable request.

## Supplementary Materials

The following supporting information can be downloaded at: https://www.mdpi.com/article/10.3390/vaccines14100836/s1, Table S1: PRISMA study selection; Table S2: Trial reports and overlap structure; Table S3: Outcome-specific risk-of-bias assessment using the revised Cochrane Risk of Bias tool for randomized trials (RoB 2); Table S4: Detailed efficacy estimates for virologically confirmed dengue; Table S5: Hospitalization estimates; Table S6: Serotype-specific efficacy; Table S7: Narrative synthesis of immunogenicity findings reported in the included randomized trial reports; Table S8: GRADE assessment of the certainty of evidence by vaccine and outcome; Table S9: Safety outcomes reported in the included randomized trial reports; Table S10: Comparison of previous dengue vaccine evidence syntheses and complementary evidence with the present review; Figure S1: Summary of outcome-specific risk-of-bias judgements using RoB 2; Figure S2: Traffic-light plot of outcome-specific risk-of-bias judgements using RoB 2; Figure S3: CYD-TDV efficacy against virologically confirmed dengue; Figure S4: TAK-003 efficacy and follow-up estimates against virologically confirmed dengue; Figure S5: CYD-TDV serotype-specific efficacy; Figure S6: TAK-003 serotype-specific efficacy and follow-up estimates; Figure S7: CYD-TDV effect on dengue-related hospitalization; Figure S8: TAK-003 effect and follow-up estimates on dengue-related hospitalization.

## Abbreviations

The following abbreviations are used in this manuscript:

ARR	Absolute risk reduction
GRADE	Grading of Recommendations Assessment, Development and Evaluation
NNV	Number needed to vaccinate
RR	Risk ratio
RoB 2	Revised Cochrane Risk of Bias tool
CI	Confidence interval
CYD-TDV	Chimeric yellow fever dengue–tetravalent dengue vaccine
DENV	Dengue virus
DENV-1	Dengue virus serotype 1
DENV-2	Dengue virus serotype 2
DENV-3	Dengue virus serotype 3
DENV-4	Dengue virus serotype 4
GMR	Geometric mean ratio
GMT	Geometric mean titre
I^2^	Inconsistency statistic
PRISMA	Preferred Reporting Items for Systematic Reviews and Meta-Analyses
PROSPERO	International Prospective Register of Systematic Reviews
TAK-003	Tetravalent dengue vaccine candidate
VCD	Virologically confirmed dengue

## References

[1] Zhang W.X., Zhao T.Y., Wang C.C., He Y., Lu H.Z., Zhang H.T., Wang L.M., Zhang M., Li C.X., Deng S.Q. (2025). Assessing the Global Dengue Burden: Incidence, Mortality, and Disability Trends over Three Decades. PLoS Negl. Trop. Dis..

[2] Deng J., Zhang H., Wang Y., Liu Q., Du M., Yan W., Qin C., Zhang S., Chen W., Zhou L. (2024). Global, Regional, and National Burden of Dengue Infection in Children and Adolescents: An Analysis of the Global Burden of Disease Study 2021. eClinicalMedicine.

[3] Cattarino L., Rodriguez-Barraquer I., Imai N., Cummings D.A.T., Ferguson N.M. (2020). Mapping Global Variation in Dengue Transmission Intensity. Sci. Transl. Med..

[4] Lim A., Shearer F.M., Sewalk K., Pigott D.M., Clarke J., Ghouse A., Judge C., Kang H., Messina J.P., Kraemer M.U.G. (2025). The Overlapping Global Distribution of Dengue, Chikungunya, Zika and Yellow Fever. Nat. Commun..

[5] Childs M.L., Lyberger K., Harris M.J., Burke M., Mordecai E.A. (2025). Climate Warming Is Expanding Dengue Burden in the Americas and Asia. Proc. Natl. Acad. Sci. USA.

[6] Narvaez F., Montenegro C., Juarez J.G., Zambrana J.V., Gonzalez K., Videa E., Arguello S., Barrios F., Ojeda S., Plazaola M. (2025). Dengue Severity by Serotype and Immune Status in 19 Years of Pediatric Clinical Studies in Nicaragua. PLoS Negl. Trop. Dis..

[7] Biswal S., Borja-Tabora C., Martinez Vargas L., Velásquez H., Theresa Alera M., Sierra V., Johana Rodriguez-Arenales E., Yu D., Wickramasinghe V.P., Duarte Moreira E. (2020). Efficacy of a Tetravalent Dengue Vaccine in Healthy Children Aged 4-16 Years: A Randomised, Placebo-Controlled, Phase 3 Trial. Lancet.

[8] López-Medina E., Biswal S., Saez-Llorens X., Borja-Tabora C., Bravo L., Sirivichayakul C., Vargas L.M., Alera M.T., Velásquez H., Reynales H. (2022). Efficacy of a Dengue Vaccine Candidate (TAK-003) in Healthy Children and Adolescents 2 Years after Vaccination. J. Infect. Dis..

[9] Rivera L., Biswal S., Sáez-Llorens X., Reynales H., López-Medina E., Borja-Tabora C., Bravo L., Sirivichayakul C., Kosalaraksa P., Martinez Vargas L. (2022). Three-Year Efficacy and Safety of Takeda’s Dengue Vaccine Candidate (TAK-003). Clin. Infect. Dis..

[10] Tricou V., Yu D., Reynales H., Biswal S., Saez-Llorens X., Sirivichayakul C., Lopez P., Borja-Tabora C., Bravo L., Kosalaraksa P. (2024). Long-Term Efficacy and Safety of a Tetravalent Dengue Vaccine (TAK-003): 4·5-Year Results from a Phase 3, Randomised, Double-Blind, Placebo-Controlled Trial. Lancet Glob. Health.

[11] Borja-Tabora C., Fernando L., Lopez Medina E., Reynales H., Rivera L., Saez-Llorens X., Sirivichayakul C., Yu D., Folschweiller N., Moss K.J. (2025). Immunogenicity, Safety, and Efficacy of a Tetravalent Dengue Vaccine in Children and Adolescents: An Analysis by Age Group. Clin. Infect. Dis..

[12] Flacco M.E., Bianconi A., Cioni G., Fiore M., Calò G.L., Imperiali G., Orazi V., Tiseo M., Troia A., Rosso A. (2024). Immunogenicity, Safety and Efficacy of the Dengue Vaccine TAK-003: A Meta-Analysis. Vaccines.

[13] Dayan G.H., Langevin E., Forrat R., Zambrano B., Noriega F., Frago C., Bouckenooghe A., Machabert T., Savarino S., DiazGranados C.A. (2020). Efficacy after 1 and 2 Doses of CYD-TDV in Dengue Endemic Areas by Dengue Serostatus. Vaccine.

[14] DiazGranados C.A., Langevin E., Bonaparte M., Sridhar S., Machabert T., Dayan G., Forrat R., Savarino S. (2021). CYD Tetravalent Dengue Vaccine Performance by Baseline Immune Profile (Monotypic/Multitypic) in Dengue-Seropositive Individuals. Clin. Infect. Dis..

[15] Forrat R., Dayan G.H., Diaz Granados C.A., Bonaparte M., Laot T., Capeding M.R., Sanchez L., Coronel D.L., Reynales H., Chansinghakul D. (2021). Analysis of Hospitalized and Severe Dengue Cases Over the 6 Years of Follow-up of the Tetravalent Dengue Vaccine (CYD-TDV) Efficacy Trials in Asia and Latin America. Clin. Infect. Dis..

[16] Godói I.P., Lemos L.L.P., de Araújo V.E., Bonoto B.C., Godman B., Guerra Júnior A.A. (2017). CYD-TDV Dengue Vaccine: Systematic Review and Meta-Analysis of Efficacy, Immunogenicity and Safety. J. Comp. Eff. Res..

[17] Andrade N.F., Molinari M.E., Bernardi J.C., David G.B., Leandro G.O., Ramos N. (2026). IMMUNOGENICITY OF THE CYD-TDV VACCINE IN CHILDREN AND ADOLESCENTS: SYSTEMATIC REVIEW AND META-ANALYSIS OF RANDOMIZED CLINICAL TRIALS. Braz. J. Infect. Dis..

[18] Malisheni M., Khaiboullina S.F., Rizvanov A.A., Takah N., Murewanhema G., Bates M. (2017). Clinical Efficacy, Safety, and Immunogenicity of a Live Attenuated Tetravalent Dengue Vaccine (CYD-TDV) in Children: A Systematic Review with Meta-Analysis. Front. Immunol..

[19] Rosa B.R., Cunha A.J., Medronho R.D. (2019). Efficacy, Immunogenicity and Safety of a Recombinant Tetravalent Dengue Vaccine (CYD-TDV) in Children Aged 2-17 Years: Systematic Review and Meta-Analysis. BMJ Open.

[20] Bengolea A., Scigliano C., Ramos-Rojas J.T., Rada G., Catalano H.N., Izcovich A. (2024). Effectiveness and Safety of the Tetravalent TAK-003 Dengue Vaccine: A Systematic Review. Medicina.

[21] Pizzol D., Rahmati M., Malakoutinia F., Boyer L., Fond G., Caminada S., Yon D.K., Shin J.I., Gawronska J., Gomez G. (2026). Evaluating the Safety and Efficacy of Dengue Vaccines: A Comprehensive Systematic Review and Meta-Analysis. Acta Trop..

[22] Kumbhare M.R., Chandak S.M., Dukare A.B., Kshatriya V.V., Velhal S.S. (2025). An Updated Comprehensive Review of the Dengue Vaccine: Development, Mechanism, Efficacy, and Safety. J. Bio-X Res..

[23] Okoye E.C., Mitra A.K., Lomax T., Nunaley C. (2024). Dengue Fever Epidemics and the Prospect of Vaccines: A Systematic Review and Meta-Analysis Using Clinical Trials in Children. Diseases.

[24] Naderian R., Eslami M., Ahmad S., Paraandavaji E., Yaghmayee S., Soltanipur M., Naderian R., Pajand O., Tajdini P., Alizadeh A. (2025). Efficacy, Immune Response, and Safety of Dengue Vaccines in Adolescents: A Systematic Review. Rev. Med. Virol..

[25] Wang R., Kim B., Mishra H., Kain K.C. (2025). Advancing Dengue Vaccine Development: Challenges, Innovations, and the Path toward Global Protection. Pediatr. Investig..

[26] Kant R., Singh A., Ali A.B., Nagre S., Ganguly N.K., Rana R. (2025). Dengue Vaccine Effectiveness and Safety: A Systematic Analysis of Recent Clinical Trial Data. Virol. J..

[27] Wilder-Smith A., Cherian T., Hombach J. (2025). Dengue Vaccine Development and Deployment into Routine Immunization. Vaccines.

[28] Centers for Disease Control and Prevention Dengue Vaccine. https://www.cdc.gov/dengue/hcp/vaccine/index.html.

[29] Hassan J., Toh T.-H., Sivapunniam S.K., Hasim R., Ghazali N.F., Sulaiman S., Koh M.T., Meyer S., Toh M.-L., Zocchetti C. (2021). Immunogenicity and Safety of a Tetravalent Dengue Vaccine Administered Concomitantly or Sequentially with Quadrivalent Human Papillomavirus Vaccine in Boys and Girls 9–13 Years of Age in Malaysia. Pediatr. Infect. Dis. J..

[30] El Hindi T., Anugulruengkitt S., Lapphra K., Limkittikul K., Tangsathapornpong A., Galindo-Tsoukas C., Hellwig M., Roubinis N., Schuring R., Biswal S. (2025). Immunogenicity and Safety of the Live-Attenuated Tetravalent Dengue Vaccine (TAK-003) Co-Administered with Recombinant 9-Valent Human Papillomavirus Vaccine. Vaccine.

[31] Hss A.-S., Koh M.-T., Tan K.K., Chan L.G., Zhou L., Bouckenooghe A., Crevat D., Hutagalung Y. (2013). Safety and Immunogenicity of a Tetravalent Dengue Vaccine in Healthy Children Aged 2–11 Years in Malaysia: A Randomized, Placebo-Controlled, Phase III Study. Vaccine.

[32] Capeding M.R., Tran N.H., Hadinegoro S.R.S., Ismail H.I.H.J.M., Chotpitayasunondh T., Chua M.N., Luong C.Q., Rusmil K., Wirawan D.N., Nallusamy R. (2014). Clinical Efficacy and Safety of a Novel Tetravalent Dengue Vaccine in Healthy Children in Asia: A Phase 3, Randomised, Observer-Masked, Placebo-Controlled Trial. Lancet.

[33] Villar L., Dayan G.H., Arredondo-García J.L., Rivera D.M., Cunha R., Deseda C., Reynales H., Costa M.S., Morales-Ramírez J.O., Carrasquilla G. (2015). Efficacy of a Tetravalent Dengue Vaccine in Children in Latin America. N. Engl. J. Med..

[34] Biswal S., Mendez Galvan J.F., Macias Parra M., Galan-Herrera J.-F., Carrascal Rodriguez M.B., Rodriguez Bueno E.P., Brose M., Rauscher M., LeFevre I., Wallace D. (2021). Immunogenicity and Safety of a Tetravalent Dengue Vaccine in Dengue-Naïve Adolescents in Mexico City. Rev. Panam. Salud Publica.

[35] Sáez-Llorens X., Biswal S., Borja-Tabora C., Fernando L., Liu M., Wallace D., Folschweiller N., Reynales H., LeFevre I., TIDES Study Group (2023). Effect of the Tetravalent Dengue Vaccine TAK-003 on Sequential Episodes of Symptomatic Dengue. Am. J. Trop. Med. Hyg..

[36] Biswal S., Reynales H., Saez-Llorens X., Lopez P., Borja-Tabora C., Kosalaraksa P., Sirivichayakul C., Watanaveeradej V., Rivera L., Espinoza F. (2019). Efficacy of a Tetravalent Dengue Vaccine in Healthy Children and Adolescents. N. Engl. J. Med..

[37] Sridhar S., Luedtke A., Langevin E., Zhu M., Bonaparte M., Machabert T., Savarino S., Zambrano B., Moureau A., Khromava A. (2018). Effect of Dengue Serostatus on Dengue Vaccine Safety and Efficacy. N. Engl. J. Med..

[38] Simmons C.P., Farrar J.J., van Vinh Chau N., Wills B. (2012). Dengue. N. Engl. J. Med..

[39] Centers for Disease Control and Prevention Vaccine Eligibility & Recommendations. https://archive.cdc.gov/www_cdc_gov/dengue/hcp/vaccine/eligibility.html.

[40] World Health Organization WHO Position Paper on Dengue Vaccines, May 2024. https://www.who.int/publications/i/item/who-wer-9918-203-224.

[41] White L.J., Young E.F., Stoops M.J., Henein S.R., Adams E.C., Baric R.S., de Silva A.M. (2021). Defining Levels of Dengue Virus Serotype-Specific Neutralizing Antibodies Induced by a Live Attenuated Tetravalent Dengue Vaccine (TAK-003). PLoS Negl. Trop. Dis..

[42] DeMaso C.R., Karwal L., Zahralban-Steele M., Dominguez D., Springer Z.-L., Kaiser M., Palani S., Rindfleisch T., Bohning K., Hather G. (2022). Specificity and Breadth of the Neutralizing Antibody Response to a Live-Attenuated Tetravalent Dengue Vaccine. J. Infect. Dis..

[43] Sirivichayakul C., Barranco-Santana E.A., Rivera I.E., Kilbury J., Raanan M., Borkowski A., Papadimitriou A., Wallace D. (2022). Long-Term Safety and Immunogenicity of a Tetravalent Dengue Vaccine Candidate in Children and Adults: A Randomized, Placebo-Controlled, Phase 2 Study. J. Infect. Dis..

[44] Hadinegoro S.R., Arredondo-García J.L., Capeding M.R., Deseda C., Chotpitayasunondh T., Dietze R., Ismail H.I.H.M., Reynales H., Limkittikul K., Rivera-Medina D.M. (2015). Efficacy and Long-Term Safety of a Dengue Vaccine in Regions of Endemic Disease. N. Engl. J. Med..

[45] World Health Organization Vaccines and Immunization: Dengue. https://www.who.int/news-room/questions-and-answers/item/dengue-vaccines.

[46] Arce-Huamani M.A., Carrascal-Astola W., Haro-Vásquez B.C., Navarro-Ochoa B., Chuquihuara-Guerrero K.M., Pineda-Chuquiyauri A.M., Paucar-Sanchez L.C., Ortiz-Arica M.M. (2026). Behavioral, Sociocultural, and Institutional Barriers to Dengue Prevention and Control Among Rural Communities in the Peruvian Amazon. Healthcare.

[47] Gianguzzo V.M., Rottura M., Sacco F.M., Donadu M.G., Sanna G., Irrera N., Imbalzano E., Arcoraci V., Melita G., Marino Y. (2026). Post-Marketing Safety Profile of Dengue Vaccines CYD-TDV and TAK-003: Analysis of Adverse Event Reports from a European Database. Front. Pharmacol..

