# Supplementary material for: Efficacy, Immunogenicity, Safety, and Evidence Independence of CYD-TDV and TAK-003 Dengue Vaccines in Children and Adolescents: A Systematic Review and Meta-Analysis
Source: Vaccines (Basel). 2026 Sep 22;14(10):836. doi: 10.3390/vaccines14100836 (PMC13709451; doi:10.3390/vaccines14100836)
Supplement: Supplementary file 1 [file vaccines-14-00836-s001.zip › vaccines-4514018-supplementary.pdf]

Supplementary Table S1. PRISMA study selection

PRISMA stage	Records/reports, n	Details
**Records identified**	1,462	Cochrane, n = 210; Embase, n = 82; PubMed, n = 133; Scopus, n = 828; Web of Science, n = 209
**Duplicate records removed**	404	Removed before screening
**Unique records screened**	1,058	Title and abstract screening
**Records excluded after screening**	1,042	Excluded after title/abstract review
**Full-text reports sought**	16	Reports selected for full-text assessment
**Reports not retrieved**	0	All reports were retrieved
**Full-text reports assessed**	16	Eligibility assessment
**Full-text reports excluded**	7	Overlapping populations with shorter follow-up or no additional relevant outcome information, n = 3; wrong target population, n = 2; did not meet the inclusion criteria, n = 2
**Randomized trial and related follow-up reports included**	9	Final reports included in the systematic review

Supplementary Table S2. Trial reports and overlap structure

Vaccine	Report	Trial/program structure	Population overlap	Recommended analytic role
**CYD-TDV**	Hss AS et al. (2013) [31]	Immunogenicity/safety report	Independent from efficacy trials	Narrative/supplementary immunogenicity
**CYD-TDV**	Capeding et al. (2014) [32]	Asian efficacy trial	Independent CYD-TDV trial	Main CYD-TDV efficacy and hospitalization meta-analysis
**CYD-TDV**	Villar et al. (2015) [33]	Latin American efficacy trial	Independent CYD-TDV trial	Main CYD-TDV efficacy and hospitalization meta-analysis
**CYD-TDV**	Forrat et al. (2021) [15]	Post hoc six-year follow-up of CYD14, CYD15, and CYD23/57	Overlaps with the CYD-TDV efficacy trial populations reported by Capeding et al. [32] and Villar et al. [33]; additionally includes the phase IIb CYD23/57 programme	Narrative synthesis of hospitalized and severe VCD by baseline serostatus and age; long-term safety and GRADE assessment; not pooled as independent evidence

**TAK-003**	Biswal et al. (2020) [7]	Pivotal efficacy report	Core TAK-003 trial population	Main TAK-003 efficacy and hospitalization estimate
**TAK-003**	Biswal et al. (2021) [34]	Adolescent immunogenicity/safety report	Separate small immunogenicity-focused population	Narrative/supplementary immunogenicity
**TAK-003**	Lopez et al. (2022) [8]	Follow-up report	Overlaps with TAK-003 pivotal trial	Descriptive follow-up only
**TAK-003**	Sáez et al. (2023) [35]	Secondary/follow-up analysis	Overlaps with TAK-003 pivotal trial	Descriptive/supplementary only
**TAK-003**	Tricou et al. (2024) [10]	Long-term follow-up report	Overlaps with TAK-003 pivotal trial	Long-term descriptive synthesis only

Note: Forrat et al. (2021) [15] combined long-term follow-up data from the CYD14, CYD15, and CYD23/57 trial programmes. Because CYD14 and CYD15 participants overlapped with the populations reported by Capeding et al. (2014) [32] and Villar et al. (2015) [33], the Forrat report was retained for severe dengue, baseline-serostatus, age-related, and long-term safety analyses but was not treated as statistically independent in pooled efficacy or hospitalization estimates.

Supplementary Table S3. Outcome-specific risk-of-bias assessment using the revised Cochrane Risk of Bias tool for randomized trials (RoB 2)

**Report**	**Result assessed**	**D1: Randomization process**	**D2: Deviations from intended interventions**	**D3: Missing outcome data**	**D4: Measurement of the outcome**	**D5: Selection of the reported result**	**Overall risk of bias**	**Main rationale**
**Hss AS et al. (2013) [31]**	Immunogenicity	Low risk	Some concerns	Low risk	Low risk	Low risk	Some concerns	Randomization and allocation procedures were considered adequate, and immunogenicity was measured using objective laboratory methods. Some concerns remained because blinding of participants and

								personnel was not described with sufficient detail.
**Hss AS et al. (2013) [31]**	Safety	Low risk	Some concerns	Low risk	Some concerns	Low risk	Some concerns	The placebo-controlled design reduced the likelihood of systematic differences between groups. However, incomplete reporting of participant and personnel blinding could have influenced the reporting of subjective adverse events.
**Capeding et al. (2014) [32]**	Virologically confirmed dengue, hospitalization, and serotype-specific efficacy	Low risk	Low risk	Low risk	Low risk	Low risk	Low risk	Randomization, allocation concealment, masking, outcome ascertainment, and reporting were considered adequate. Virologically confirmed and hospitalized dengue were assessed using prespecified and clinically objective criteria.
**Capeding et al. (2014) [32]**	Safety	Low risk	Low risk	Low risk	Low risk	Low risk	Low risk	Safety surveillance was conducted in both randomized groups using prespecified reporting procedures, with no important concern identified across the five RoB 2 domains.
**Villar et al. (2015) [33]**	Virologically confirmed dengue, hospitalization, and	Low risk	Low risk	Low risk	Low risk	Low risk	Low risk	The randomized, observer-masked, placebo-controlled design and objective

	serotype-specific efficacy							virological confirmation supported a low risk-of-bias judgement for the efficacy outcomes.
**Villar et al. (2015) [33]**	Safety	Low risk	Low risk	Low risk	Low risk	Low risk	Low risk	Safety outcomes were monitored prospectively in both randomized groups, and no substantial concern regarding missing data, outcome measurement, or selective reporting was identified.
**Forrat et al. (2021) [15]**	Hospitalized VCD, severe VCD, and long-term safety by baseline serostatus and age	Low risk	Some concerns	Some concerns	Low risk	Some concerns	Some concerns	The source trials were randomized and placebo-controlled, and hospitalized and severe VCD were assessed using objective virological and clinical criteria. However, this was a post hoc analysis that combined phase III and phase IIb trial populations, used several measured or inferred methods to determine baseline serostatus, and analyzed participants who received at least one injection. Some concerns also remained because of incomplete long-term follow-up in one trial

								and the availability of multiple eligible analytical methods and subgroup definitions. Multiple imputation and sensitivity analyses reduced, but did not eliminate, these concerns.
**Biswal et al. (2020) [7]**	Virologically confirmed dengue, hospitalization, and serotype-specific efficacy	Low risk	Low risk	Low risk	Low risk	Low risk	Low risk	The randomized, double-blind, placebo-controlled design, prespecified analyses, and laboratory confirmation of dengue supported a low risk-of-bias judgement.
**Biswal et al. (2020) [7]**	Safety	Low risk	Low risk	Low risk	Low risk	Low risk	Low risk	Safety surveillance and serious adverse-event reporting were conducted prospectively in both treatment groups, with no important methodological concern identified.
**Biswal et al. (2021) [34]**	Immunogenicity and safety	Low risk	Low risk	Low risk	Low risk	Low risk	Low risk	Randomization and masking were considered adequate. Immunogenicity was measured through objective neutralization assays, and safety outcomes were assessed prospectively in the vaccine and placebo groups.
**Lopez et al. (2022) [8]**	Virologically confirmed dengue, hospitalization,	Low risk	Low risk	Low risk	Low risk	Low risk	Low risk	This prespecified follow-up retained the randomized comparison and used

	immunogenicity, and safety							objective outcome definitions. Overlap with the primary trial report was addressed during evidence synthesis and was not considered a risk-of-bias domain.
**Sáez et al. (2023) [35]**	Sequential symptomatic dengue episodes	Low risk	Low risk	Low risk	Low risk	Some concerns	Some concerns	The analysis was based on a randomized trial population and objectively confirmed dengue episodes. Some concerns remained because this was a secondary analysis of sequential outcomes, increasing uncertainty regarding selection among multiple eligible analyses.
**Tricou et al. (2024) [10]**	Virologically confirmed dengue, hospitalization, serotype-specific efficacy, and long-term safety	Low risk	Low risk	Low risk	Low risk	Low risk	Low risk	The long-term follow-up preserved the randomized comparison and used prespecified, objectively measured clinical outcomes. Overlapping participants were considered in the synthesis but did not independently increase risk of bias within the trial result.

Note: D1, bias arising from the randomization process; D2, bias due to deviations from the intended interventions; D3, bias due to missing outcome data; D4, bias in the measurement of the outcome; D5, bias in the selection of the reported result. Assessments addressed the effect of assignment to the intervention. Results were grouped within a report only when the same domain-level judgements applied to all listed outcomes. Sponsor or manufacturer involvement was

recorded separately as a contextual funding consideration and was not treated as an additional RoB 2 domain or as an automatic reason for downgrading a result. Detailed responses to the RoB 2 signalling questions were completed independently by two reviewers and reconciled by consensus.

Supplementary Table S4. Detailed efficacy estimates for virologically confirmed dengue

Vaccine	Report	Analytic role	Vaccine events/total	Control events/total	Risk ratio	95% CI
**CYD-TDV**	Capeding et al. (2014) [32]	Main pooled CYD-TDV estimate	117/6,526	133/3,227	0.43	0.34–0.56
**CYD-TDV**	Villar et al. (2015) [33]	Main pooled CYD-TDV estimate	176/11,793	221/5,809	0.39	0.32–0.48
**CYD-TDV**	Pooled estimate	Main pooled estimate	293/18,319	354/9,036	0.41	0.35–0.48
**TAK-003**	Biswal et al. (2020) [7]	Main TAK-003 estimate	114/12,700	206/6,316	0.28	0.22–0.35
**TAK-003**	Lopez et al. (2022) [8]	Follow-up; not pooled with primary report	97/12,577	106/6,268	0.46	0.35–0.60
**TAK-003**	Sáez et al. (2023) [35]	Secondary/follow-up outcome; not pooled	5/13,380	13/6,687	0.19	0.07–0.54
**TAK-003**	Tricou et al. (2024) [10]	Long-term follow-up; not pooled with primary report	442/13,380	547/6,687	0.40	0.36–0.46

Note: TAK-003 follow-up reports should not be pooled together as independent studies because they include overlapping participants from the same clinical trial programme.

Supplementary Table S5. Hospitalization estimates

Vaccine	Report	Analytic role	Vaccine events/total	Control events/total	Risk ratio	95% CI

**CYD-TDV**	Capeding et al. (2014) [32]	Main pooled CYD-TDV estimate	40/6,848	61/3,424	0.33	0.22–0.49
**CYD-TDV**	Villar et al. (2015) [33]	Main pooled CYD-TDV estimate	17/281	43/389	0.55	0.32–0.94
**CYD-TDV**	Pooled estimate	Main pooled estimate	57/7,129	104/3,813	0.41	0.25–0.67
**TAK-003**	Biswal et al. (2020) [7]	Main TAK-003 estimate	13/12,700	88/6,316	0.10	0.05–0.18
**TAK-003**	Lopez et al. (2022) [8]	Follow-up; not pooled with primary report	11/12,577	22/6,268	0.25	0.12–0.51
**TAK-003**	Tricou et al. (2024) [10]	Long-term follow-up; not pooled with primary report	46/13,380	142/6,687	0.16	0.12–0.23

Note: Hospitalization denominators reflect the population/time-at-risk reported for the hospitalization endpoint in each trial report and may differ from denominators used for virologically confirmed dengue efficacy. TAK-003 hospitalization estimates from repeated follow-up reports should be displayed descriptively, not pooled as independent evidence.

Supplementary Table S6. Serotype-specific efficacy

**Vaccine**	**Report/analysis**	**Serotype**	**Vaccine events/total**	**Control events/total**	**Risk ratio**	**95% CI**	**Analytic interpretation**

**CYD-TDV**	Pooled by serotype	DENV-1	117/19,026	116/9,406	0.50	0.39–0.64	Valid pooled serotype-specific estimate
**CYD-TDV**	Pooled by serotype	DENV-2	96/19,056	79/9,472	0.60	0.45–0.81	Valid pooled serotype-specific estimate
**CYD-TDV**	Pooled by serotype	DENV-3	53/19,127	105/9,494	0.25	0.18–0.35	Valid pooled serotype-specific estimate
**CYD-TDV**	Pooled by serotype	DENV-4	35/19,127	74/9,471	0.23	0.16–0.35	Valid pooled serotype-specific estimate
**TAK-003**	Biswal et al. (2020) [7]	DENV-1	38/12,700	82/6,316	0.30	0.20–0.46	Main TAK-003 serotype-specific estimate
**TAK-003**	Biswal et al. (2020) [7]	DENV-2	8/12,700	80/6,316	0.05	0.02–0.10	Main TAK-003 serotype-specific estimate
**TAK-003**	Biswal et al. (2020) [7]	DENV-3	63/12,700	60/6,316	0.52	0.37–0.74	Main TAK-003 serotype-specific estimate
**TAK-003**	Biswal et al. (2020) [7]	DENV-4	5/12,700	5/6,316	0.50	0.14–1.72	Imprecise estimate
**TAK-003**	Lopez et al. (2022) [8]	DENV-1	41/12,577	49/6,268	0.42	0.28–0.63	Follow-up; not pooled
**TAK-003**	Lopez et al. (2022) [8]	DENV-2	14/12,577	27/6,268	0.26	0.14–0.49	Follow-up; not pooled
**TAK-003**	Lopez et al. (2022) [8]	DENV-3	14/12,577	26/6,268	0.27	0.14–0.51	Follow-up; not pooled
**TAK-003**	Lopez et al. (2022) [8]	DENV-4	5/12,577	4/6,268	0.62	0.17–2.32	Follow-up; imprecise; not pooled
**TAK-003**	Tricou et al. (2024) [10]	DENV-1	222/13,377	230/6,686	0.48	0.40–0.58	Long-term follow-up; not pooled
**TAK-003**	Tricou et al. (2024) [10]	DENV-2	98/13,377	193/6,686	0.18	0.13–0.23	Long-term follow-up; not pooled
**TAK-003**	Tricou et al. (2024) [10]	DENV-3	132/13,377	113/6,686	0.58	0.45–0.75	Long-term follow-up; not pooled

**TAK-003**	Tricou et al. (2024) [10]	DENV-4	24/13,377	23/6,686	0.52	0.29–0.92	Long-term follow-up; not pooled

Note: No overall cross-serotype pooled estimate should be presented because DENV-1, DENV-2, DENV-3, and DENV-4 are correlated outcomes within the same trial participants.

Supplementary Table S7. Narrative synthesis of immunogenicity findings reported in the included randomized trial reports

Vaccine	Report	Population and follow-up	Immunogenicity metric reported	Main findings	Interpretation in the present review
**CYD-TDV**	Hss AS et al. (2013) [31]	Children aged 2–11 years; three-dose schedule with follow-up through 18 months	Serotype-specific neutralizing antibody GMTs and seropositivity rates	Vaccination induced neutralizing antibody responses against all four DENV serotypes after completion of the vaccination schedule.	Findings were retained as supportive evidence. No pooled effect was calculated because the reported titre scale and follow-up time points were not directly comparable with those of the other trials.
**CYD-TDV**	Capeding et al. (2014) [32]	Children aged 2–14 years in Asia; follow-up through 25 months	Serotype-specific neutralizing antibody titres and GMTs	Neutralizing antibody responses were reported against DENV-1, DENV-2, DENV-3, and DENV-4 following vaccination.	Results were interpreted within the original trial because immunogenicity measures could not be harmonized reliably across studies or vaccine platforms.
**CYD-TDV**	Villar et al. (2015) [33]	Children and adolescents aged 9–16 years in Latin America; follow-up through 25 months	Serotype-specific neutralizing antibody titres and GMTs	Vaccination elicited antibody responses against all four DENV serotypes, although the magnitude of the responses differed by serotype.	Immunogenicity was considered complementary to clinical efficacy and was not treated as a validated surrogate of protection.
**TAK-003**	Biswal et al. (2020) [7]	Children and adolescents aged 4–16 years; primary phase 3 efficacy analysis	Serotype-specific neutralizing antibody GMTs and seropositivity rates,	Neutralizing antibody responses were observed against all four DENV serotypes. Responses differed	Findings were synthesized narratively because seropositive and seronegative groups, serotypes, and assessment time

			reported according to baseline serostatus	by serotype and baseline dengue serostatus.	points could not be represented by a single comparable effect measure.
**TAK-003**	Biswal et al. (2021) [34]	Dengue-naïve adolescents aged 12–17 years in Mexico; follow-up through 9 months	Serotype-specific neutralizing antibody GMTs and seropositivity rates	TAK-003 induced neutralizing antibody responses against the four DENV serotypes in the adolescent population studied.	This report was treated as a separate supportive immunogenicity study and was not combined quantitatively with the larger phase 3 trial programme.
**TAK-003**	Lopez et al. (2022) [8]	Children and adolescents aged 4–16 years; approximately two years of follow-up	Serotype-specific neutralizing antibody GMTs according to baseline serostatus and follow-up time	Neutralizing antibody responses persisted during follow-up but varied by serotype, baseline serostatus, and time since vaccination.	The report involved participants from the main TAK-003 trial programme and was interpreted as longitudinal follow-up rather than independent evidence.
**TAK-003**	Tricou et al. (2024) [10]	Children and adolescents aged 4–16 years; 4.5-year follow-up	Long-term serotype-specific neutralizing antibody measures	Long-term findings supported persistence of vaccine-induced immune responses, with variation according to serotype and baseline immune status.	Findings were summarized descriptively and were not pooled with earlier reports because the publications involved overlapping participants from the same trial programme.

Note: DENV, dengue virus; GMT, geometric mean titre. Immunogenicity outcomes were reported using heterogeneous assays, transformations, summary measures, participant strata, and assessment time points. Consequently, raw mean differences and standardized mean differences were not calculated. Immunogenicity findings were treated as supportive evidence and should not be interpreted as directly comparable measures of clinical efficacy or as established correlates of protection.

Supplementary Table S8. GRADE assessment of the certainty of evidence by vaccine and outcome

Outcome	Vaccine and evidence base	Certainty of evidence	Risk of bias	Inconsistency	Indirectness	Imprecision	Publication bias	Overall rationale
**Virologically confirmed dengue**	CYD-TDV: two independent randomized efficacy trials	Moderate	Not serious	Not serious	Serious	Not serious	Undetected; cannot be excluded	The two trials provided consistent placebo-controlled evidence with precise estimates. Certainty was downgraded because the aggregate estimate combined populations with different ages and baseline dengue serostatus, which limits direct application to specific eligible groups.
**Virologically confirmed dengue**	TAK-003: one primary randomized efficacy trial; overlapping follow-up reports considered descriptively	Moderate	Not serious	Not assessable	Serious	Not serious	Undetected; cannot be excluded	The primary trial showed a large and precise effect. Certainty was downgraded because evidence was derived mainly from one clinical trial programme and vaccine performance varied according to baseline serostatus and infecting serotype.
**Dengue-related hospitalization**	CYD-TDV: two independent randomized efficacy trials, supplemented	Low	Not serious	Serious	Serious	Not serious	Undetected; cannot be excluded	Certainty was downgraded because the pooled analysis showed moderate heterogeneity

	by the overlapping six-year follow-up analysis reported by Forrat et al. (2021) [15]							and the hospitalization populations and denominators differed between trials. Interpretation also depends strongly on age and baseline serostatus. The long-term follow-up provided additional subgroup-specific evidence by baseline serostatus and age but was not treated as statistically independent.
**Dengue-related hospitalization**	TAK-003: one primary randomized efficacy trial; overlapping follow-up reports considered descriptively	Moderate	Not serious	Not assessable	Serious	Not serious	Undetected; cannot be excluded	The primary trial showed a large reduction in hospitalization with a precise estimate. Certainty was downgraded because the evidence was derived mainly from one trial programme and subgroup effects differed by baseline serostatus and serotype.
**Severe dengue**	CYD-TDV: randomized efficacy trials and the overlapping six-year post hoc follow-up of CYD14, CYD15, and CYD23/57	Low	Some concerns	Not assessable	Serious	Serious	Undetected; cannot be excluded	Severe VCD was objectively defined, and long-term findings demonstrated protection among baseline-seropositive participants, particularly those aged ≥9

	reported by Forrat et al. (2021) [15]							years. Certainty was limited because severe events were uncommon, the long-term analysis was post hoc, baseline serostatus was measured or inferred using several methods, and the populations overlapped with the primary efficacy trials. The direction of effect also differed according to baseline serostatus and age.
**Severe dengue**	TAK-003: primary randomized trial and long-term follow-up reports from the same trial programme	Low	Not serious	Not assessable	Serious	Serious	Undetected; cannot be excluded	Severe dengue events were rare, resulting in limited precision. Evidence was derived from one trial programme, and clinically relevant variation remained across baseline serostatus and infecting serotype.
**Serotype-specific efficacy**	CYD-TDV: two independent randomized efficacy trials analyzed separately by serotype	Moderate	Not serious	Not serious	Serious	Not serious	Undetected; cannot be excluded	Estimates were consistent and reasonably precise within each serotype. Certainty was downgraded because serotype-specific effects may depend on local transmission patterns,

								baseline serostatus, and age distribution.
**Serotype-specific efficacy**	TAK-003: primary randomized efficacy report and overlapping follow-up analyses	Low	Not serious	Not assessable	Serious	Serious	Undetected; cannot be excluded	Protection differed across serotypes, and the DENV-4 estimate was particularly imprecise. Evidence was derived primarily from one trial programme, limiting assessment of consistency across independent studies.
**Immunogenicity**	CYD-TDV: randomized immunogenicity and efficacy reports	Low	Some concerns	Serious	Serious	Serious	Undetected; cannot be excluded	Antibody responses were reported using heterogeneous titre measures, serotypes, time points, and participant groups. Neutralizing antibody titres were considered indirect markers because no universally accepted correlate of protection exists for dengue.
**Immunogenicity**	TAK-003: randomized immunogenicity and efficacy reports	Low	Some concerns	Serious	Serious	Serious	Undetected; cannot be excluded	Immunogenicity estimates differed by baseline serostatus, serotype, and follow-up time. Measures were not sufficiently harmonized for direct comparison, and antibody titres remain indirect

								indicators of clinical protection.
**Safety**	CYD-TDV: randomized trials and the overlapping six-year follow-up analysis by Forrat et al. (2021) [15]	Low	Some concerns	Not assessable	Serious	Serious	Undetected; cannot be excluded	Short-term serious adverse-event frequencies were generally balanced between vaccine and placebo groups. However, clinically relevant long-term safety depended strongly on baseline serostatus and age. Certainty was limited by rare hospitalized and severe dengue events, post hoc subgroup analyses, inferred baseline serostatus for many participants, and overlapping trial populations.
**Safety**	TAK-003: randomized trials and long-term follow-up reports from the same trial programme	Moderate	Not serious	Not assessable	Serious	Not serious	Undetected; cannot be excluded	Large randomized cohorts and extended follow-up provided useful safety evidence. Certainty was downgraded because most long-term evidence came from one clinical trial programme and reports contained overlapping participants.

Note: Certainty ratings were assigned according to the GRADE approach. Evidence from randomized trials initially started at high certainty and was downgraded for serious concerns regarding risk of bias, inconsistency, indirectness, imprecision, or publication bias. Inconsistency was considered not assessable when an outcome was informed by one independent trial programme. Publication bias was evaluated qualitatively because fewer than 10 independent studies were available for each outcome; therefore, funnel plots and regression-based tests were not considered reliable. Sponsor or manufacturer involvement was documented as a contextual consideration but was not treated as an independent GRADE domain or as an automatic reason for downgrading certainty. Sridhar et al. (2018) [37] was used as complementary external evidence in the Discussion but was not included in the PRISMA study count, the RoB 2 assessment, or the GRADE evidence profiles

Supplementary Table S9. Safety outcomes reported in the included randomized trial reports

Vaccine	Report	Safety follow-up	Serious adverse events	Vaccine-related serious adverse events	Deaths and discontinuations	Severe dengue and long-term safety	Interpretation
**CYD-TDV**	Hss AS et al. (2013) [31]	18 months	Serious adverse events were reported, but the article did not provide a sufficiently detailed numerical comparison for all categories used in this review.	All serious adverse events were considered unrelated to vaccination.	NR	No severe dengue safety signal was identified during the reported follow-up.	CYD-TDV was generally well tolerated in children aged 2–11 years; detailed safety estimates were limited.
**CYD-TDV**	Capeding et al. (2014) [32]	25 months	A total of 647 serious adverse-event episodes were reported: 402 in the vaccine group and 245 in the placebo	No vaccine-related serious adverse-event pattern was identified; reported events	NR	Severe dengue and hospitalization outcomes favored vaccination during the efficacy follow-up, but long-term safety	The overall frequency and nature of serious adverse events were similar between groups.

			group. Serious adverse events within 28 days after vaccination occurred in 54 children (1%) and 33 children (1%), respectively.	were mainly infections and injuries expected in the study population.		according to baseline serostatus was not established in this report.	
**CYD-TDV**	Villar et al. (2015) [33]	25 months	Serious adverse events within 28 days after an injection occurred in 81 vaccine recipients (0.6%) and 40 placebo recipients (0.6%).	No concerning imbalance in vaccine-related serious adverse events was reported.	NR	The trial reported fewer severe and hospitalized virologically confirmed dengue cases among vaccine recipients; however, long-term risk according to baseline serostatus required subsequent follow-up analyses.	Short-term serious adverse-event frequencies were balanced, but the benefit–risk profile could not be interpreted independently of baseline serostatus.
**CYD-TDV**	Forrat et al. (2021) [15]	6 years	The report focused on hospitalized and severe virologically confirmed dengue rather than providing an independent overall serious adverse-event comparison.	Not analyzed as a separate conventional vaccine-related serious adverse-event endpoint.	No dengue-related deaths were reported; detailed all-cause discontinuation data were not used as an independent pooled safety outcome.	Among baseline-seropositive participants aged ≥9 years, the HR was 0.19 (95% CI: 0.12–0.30) for hospitalized VCD and 0.15 (95% CI: 0.06–0.39) for severe VCD. Protection was also observed among seropositive children aged 6–8 years. In baseline-seronegative	The findings support long-term benefit among previously exposed recipients but confirm that CYD-TDV safety is strongly dependent on baseline serostatus and age. This overlapping follow-up report was not treated as

						recipients, the risk pattern was unfavorable, particularly among those aged <9 years.	statistically independent evidence.
**TAK-003**	Biswal et al. (2020) [7]	Approximately 18 months after the second dose	Serious adverse events occurred in 3.1% of TAK-003 recipients and 3.8% of placebo recipients.	No clinically meaningful imbalance in vaccine-related serious adverse events was identified.	NR	TAK-003 reduced hospitalized and severe dengue during the reported period, with no evidence of vaccine-associated enhanced disease.	The serious adverse-event profile was similar between groups, including among baseline-seronegative participants.
**TAK-003**	Biswal et al. (2021) [34]	9 months	Detailed serious adverse-event counts were not consistently available for the safety categories prespecified in this review.	No major vaccine-related serious adverse-event signal was reported.	NR	Severe dengue was not an efficacy endpoint in this small immunogenicity-focused trial.	TAK-003 was generally well tolerated among dengue-naïve adolescents, but the sample and follow-up were insufficient to assess rare or long-term events.
**TAK-003**	Lopez et al. (2022) [8]	Approximately 27 months after the first dose	Cumulative serious adverse-event rates were 4.0% in TAK-003 recipients and 4.8% in placebo recipients. During months 16–27, serious adverse events occurred in	No serious adverse events during the second year were considered related to the investigational product or study procedures.	During months 16–27, three deaths occurred—two in the TAK-003 group and one in the placebo group—and none was considered related to vaccination. Serious adverse events led to	Four TAK-003 recipients and 11 placebo recipients experienced severe dengue or dengue hemorrhagic fever during cumulative follow-up; estimates remained imprecise	No important long-term safety signal emerged through the second year, although continued monitoring was required for rare outcomes and

			271 participants (2.0%) and 151 participants (2.3%), respectively.		withdrawal or study discontinuation in two participants in each group.	because events were rare.	serotype-specific performance.
**TAK-003**	Sáez et al. (2023) [35]	Up to 57 months	This secondary analysis did not provide an independent comprehensive serious adverse-event dataset.	Not analyzed separately.	NR	The report assessed sequential symptomatic dengue episodes using participants from the ongoing TIDES trial.	Safety findings were not treated as statistically independent because the population overlapped with the primary TAK-003 trial reports.
**TAK-003**	Tricou et al. (2024) [10]	4.5 years	Serious adverse events continued to be monitored throughout long-term follow-up; no important new safety signal was reported.	No new vaccine-related serious adverse-event pattern was identified during long-term monitoring.	Detailed outcome-specific counts were not independently pooled because the report involved the same TIDES population.	Long-term follow-up did not indicate vaccine-associated enhanced dengue; however, protection varied by baseline serostatus and serotype, especially among dengue-naïve participants.	The report supported an acceptable long-term safety profile, but it was interpreted as extended follow-up of the same trial programme rather than as independent evidence.

Note: NR, not reported numerically or not sufficiently detailed in the included report. Reports from the TAK-003 TIDES programme involved overlapping participants and follow-up periods and were therefore summarized descriptively rather than treated as independent safety datasets. The absence of a numerical value should not be interpreted as evidence that an event did not occur. Hospitalized or severe dengue was considered relevant to safety when reported according to baseline serostatus because of the theoretical concern regarding vaccine-associated enhanced disease. Forrat et al. (2021) [15] combined six-year follow-up data from CYD14, CYD15, and CYD23/57 and was therefore retained for subgroup-specific severe dengue and long-term safety interpretation but not

treated as an independent dataset in pooled analyses. Sridhar et al. (2018) [37] was cited as complementary external evidence and was not treated as an included safety report.

Supplementary Table S10. Comparison of previous dengue vaccine evidence syntheses and complementary evidence with the present review

Reference	Design and scope	Vaccine(s) and population	Main outcomes or analytic features	Relationship to the present review
**Godói et al. (2017) [16]**	Systematic review and meta-analysis	CYD-TDV; children, adolescents, and other populations represented in the available trials	Efficacy, immunogenicity, and safety	Established the efficacy, immunogenicity, and safety profile of CYD-TDV. The present review therefore does not consider these domains novel; it additionally evaluates CYD-TDV alongside TAK-003 within a pediatric placebo-controlled evidence framework and distinguishes independent trials from overlapping follow-up reports.
**Malisheni et al. (2017) [18]**	Systematic review with meta-analysis of randomized clinical trials	CYD-TDV; children aged <18 years	Overall and serotype-specific efficacy, safety, and immunogenicity	Provided an earlier quantitative pediatric synthesis of CYD-TDV across the same major evidence domains. The present review incorporates later long-term evidence, includes TAK-003, and explicitly maps reports to their underlying randomized trial populations.
**Rosa et al. (2019) [19]**	Systematic review and meta-analysis of randomized trials	CYD-TDV; children and adolescents aged 2–17 years	Overall and serotype-specific efficacy, immunogenicity, and safety	Closely overlaps with the CYD-TDV component of the present review. The present review extends the scope to TAK-003 and places greater emphasis on long-term safety according to baseline serostatus and on the independence of the underlying trial populations.
**Flacco et al. (2024) [12]**	Systematic review and meta-analysis	TAK-003; adults and children/adolescents,	Immunogenicity, virologically confirmed dengue, serious	Provides a detailed quantitative synthesis of TAK-003. The present review does not seek to

		including baseline-seropositive and baseline-seronegative populations	adverse events, dose- and serostatus-related analyses	reproduce this vaccine-specific synthesis; rather, it evaluates TAK-003 within a two-programme pediatric framework and distinguishes successive TIDES follow-up reports from independent efficacy replications.
**Okoye et al. (2024) [23]**	Systematic review and meta-analysis of clinical trials	Multiple dengue vaccines, including CYD-TDV and TAK-003; children aged 0–17 years	Vaccine efficacy, immune response, and safety	Provides a broad pediatric synthesis across dengue vaccine candidates. The present review is narrower in vaccine and comparator eligibility and focuses specifically on placebo-controlled CYD-TDV and TAK-003 evidence, with explicit handling of overlapping follow-up publications.
**Bengolea et al. (2024) [20]**	Systematic review of randomized placebo-controlled trials with meta-analysis, RoB 2, and GRADE	TAK-003; predominantly children and adolescents	Dengue infection, severe dengue, hemorrhagic fever, mortality, adverse events, relative and absolute effects, and certainty of evidence	Demonstrates that the use of absolute effects and GRADE is not unique to the present review. The additional emphasis of the present review is therefore placed on the structure and independence of the evidence across CYD-TDV and TAK-003 trial programmes and on avoiding double counting of overlapping follow-up reports.
**Kumbhare et al. (2025) [22]**	Comprehensive narrative review	Multiple dengue vaccine platforms, including CYD-TDV and TAK-003	Vaccine development, mechanisms, immunogenicity, efficacy, and safety	Provides a broad overview of dengue vaccine development and biological mechanisms. The present review addresses a narrower quantitative question based on randomized placebo-controlled evidence in children and adolescents.
**Naderian et al. (2025) [24]**	Systematic review	CYD-TDV and TAK-003; adolescents	Efficacy, immune response, safety, serotype-specific findings, and durability of immune responses	Directly addresses both vaccines in an adolescent population. The present review encompasses a broader pediatric age range and adds quantitative synthesis of placebo-

				controlled clinical outcomes together with explicit assessment of whether successive reports represent independent trial populations.
**Wang et al. (2025) [25]**	Narrative review of dengue vaccine development	CYD-TDV, TAK-003, and Butantan-DV	Efficacy, safety, antibody-dependent enhancement, serotype-specific limitations, and future vaccine development	Provides a broad biological and developmental perspective. The present review instead focuses on the quantitative interpretation and evidential structure of placebo-controlled pediatric CYD-TDV and TAK-003 trials.
**Kant et al. (2025) [26]**	Systematic review of recent clinical trial evidence	Multiple dengue vaccines, including CYD-TDV, TAK-003, and TV003/TV005	Efficacy, safety, immunogenicity, and pooled clinical trial estimates	Provides a recent broad synthesis of dengue vaccine clinical trials. The present review is restricted to CYD-TDV and TAK-003 in children and adolescents and explicitly links publications to their source trial populations before quantitative synthesis.
**Wilder-Smith et al. (2025) [27]**	Article focused on vaccine development and deployment	CYD-TDV and TAK-003; programmatic and population-level perspective	Efficacy, safety, vaccine-enhanced disease, WHO recommendations, implementation, and routine immunization	Provides complementary programmatic and policy context rather than a trial-level meta-analysis. The present review focuses on randomized pediatric evidence and the distinction between longer follow-up and independent replication.
**Andrade et al. (2026) [17]**	Systematic review and meta-analysis of randomized clinical trials	CYD-TDV; children and adolescents	Serotype-specific neutralizing antibody responses and immunogenicity	Focuses specifically on CYD-TDV immunogenicity. The present review treats immunogenicity as supportive evidence while additionally evaluating clinical efficacy, hospitalization, severe dengue, safety, and TAK-003.
**Pizzol et al. (2026) [21]**	Comprehensive systematic review and	Multiple dengue vaccines and populations; 42 studies	Vaccine efficacy, viremia, hemorrhagic fever, immunogenicity, adverse	Provides a substantially broader synthesis across vaccines, populations, and study designs. The present review is more narrowly

	meta-analysis with GRADE		events, and certainty of evidence	restricted to pediatric placebo-controlled CYD-TDV and TAK-003 evidence and emphasizes the independence and overlap structure of the contributing randomized trial populations.
**Gianguzzo et al. (2026) [47]**	Retrospective post-marketing pharmacovigilance study; not a systematic review	CYD-TDV and TAK-003; spontaneous adverse-event reports in EudraVigilance	Post-marketing adverse drug reactions, seriousness, outcomes, and disproportionality analyses	Provides complementary real-world safety evidence outside the randomized placebo-controlled eligibility criteria of the present review. It is relevant to external safety interpretation but was not eligible for inclusion in the trial meta-analysis.
**Present review**	Systematic review and meta-analysis of randomized placebo-controlled trials and related follow-up reports	CYD-TDV and TAK-003; children and adolescents	VCD, hospitalization, severe dengue, serotype-specific efficacy, immunogenicity, short- and long-term safety, absolute effects, RoB 2, GRADE, and trial-population overlap	The contribution is incremental rather than the identification of a new vaccine effect. Successive publications are mapped to their source randomized trial populations; overlapping follow-up reports are not treated as independent efficacy replications; and relative and absolute effects are interpreted within each vaccine programme rather than as head-to-head comparisons.

Note: CYD-TDV, chimeric yellow fever–dengue tetravalent vaccine; DENV, dengue virus; GRADE, Grading of Recommendations Assessment, Development and Evaluation; RoB 2, revised Cochrane Risk of Bias tool for randomized trials; TAK-003, tetravalent dengue vaccine candidate; TIDES, Tetravalent Immunization against Dengue Efficacy Study; VCD, virologically confirmed dengue. The table is intended to clarify the scope of previous evidence syntheses and complementary evidence rather than to imply methodological superiority of the present review. Absolute effects have been reported in previous evidence syntheses, including Bengolea et al. [20]; therefore, their calculation is not presented as a novel methodological contribution. The distinctive emphasis of the present review is the explicit mapping of successive publications to their source randomized trial populations and the separation of extended follow-up from independent replication.

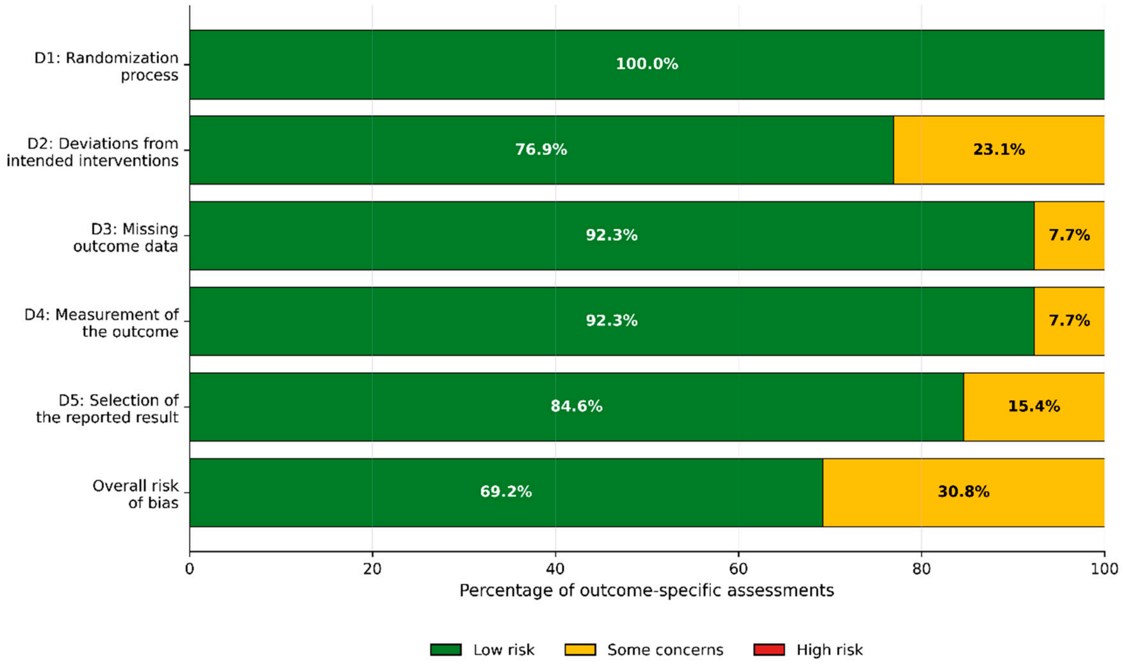

Supplementary Figure S1. Summary of outcome-specific risk-of-bias judgements using RoB 2.

The figure shows the proportion of outcome-specific assessments classified as low risk of bias, some concerns, or high risk of bias across the five RoB 2 domains and the overall judgement. Thirteen outcome-specific assessments were included. No result was judged to have a high risk of bias. D1, bias arising from the randomization process; D2, bias due to deviations from the intended interventions; D3, bias due to missing outcome data; D4, bias in the measurement of the outcome; D5, bias in the selection of the reported result.

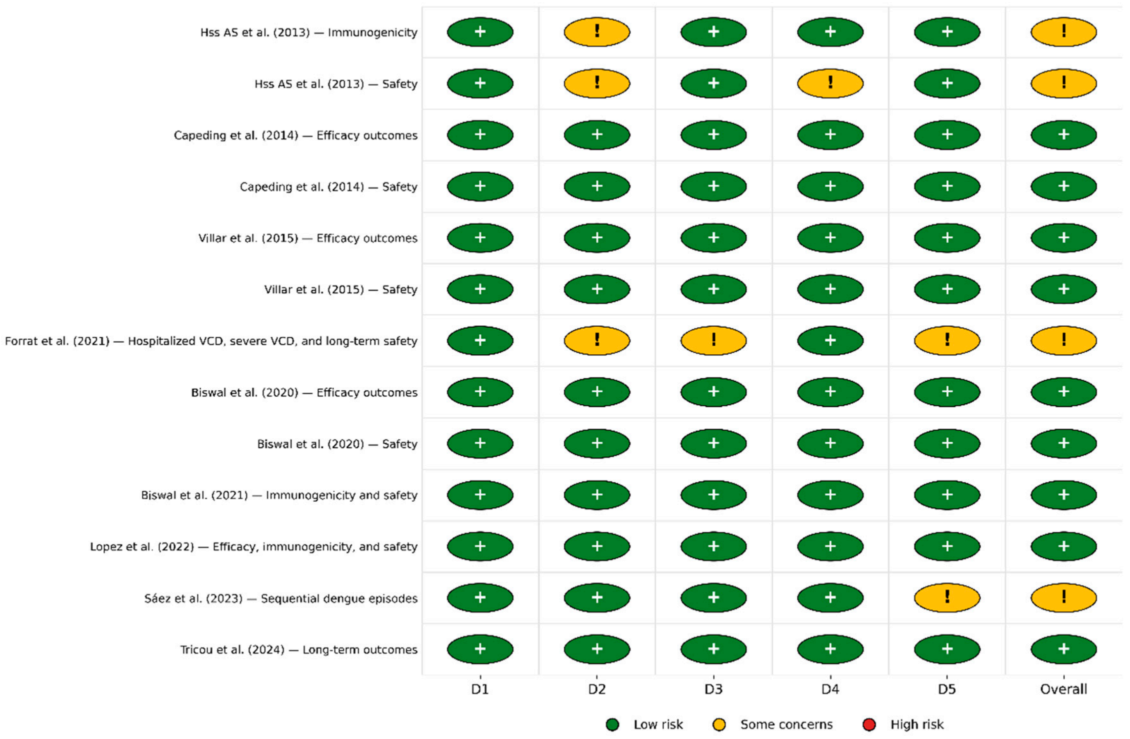

Supplementary Figure S2. Traffic-light plot of outcome-specific risk-of-bias judgements using RoB 2.

Each row represents an outcome-specific assessment from an included randomized trial or related follow-up report. Green circles with a plus sign indicate low risk of bias, yellow circles with an exclamation mark indicate some concerns, and red circles with a minus sign indicate high risk of bias. D1, bias arising from the randomization process; D2, bias due to deviations from the intended interventions; D3, bias due to missing outcome data; D4, bias in the measurement of the outcome; D5, bias in the selection of the reported result.

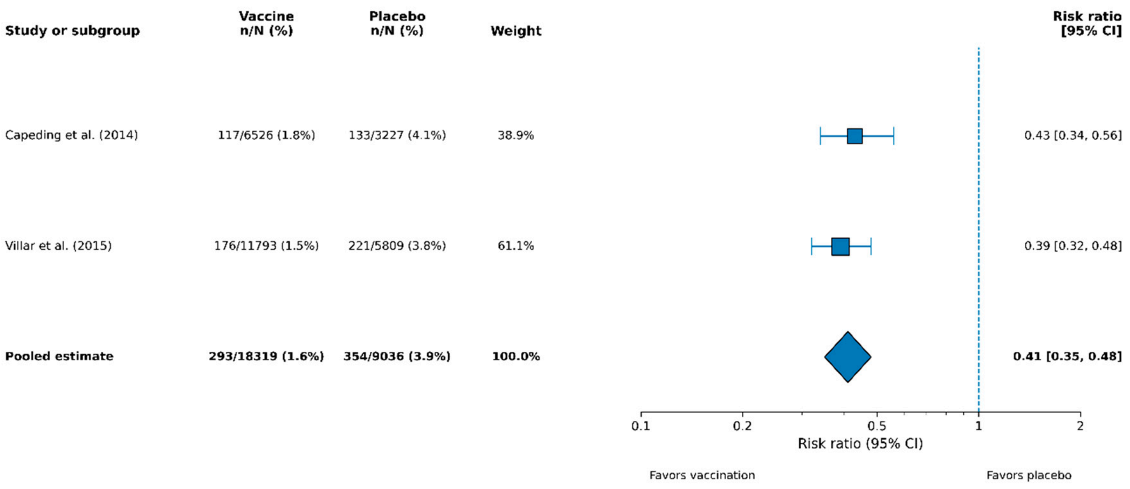

Supplementary Figure S3. CYD-TDV efficacy against virologically confirmed dengue.

The plot includes two independent randomized efficacy trials and their pooled estimate. Event counts, denominators, absolute event percentages, study weights, risk ratios, and 95% confidence intervals are shown. Risk ratios below 1 favor CYD-TDV.

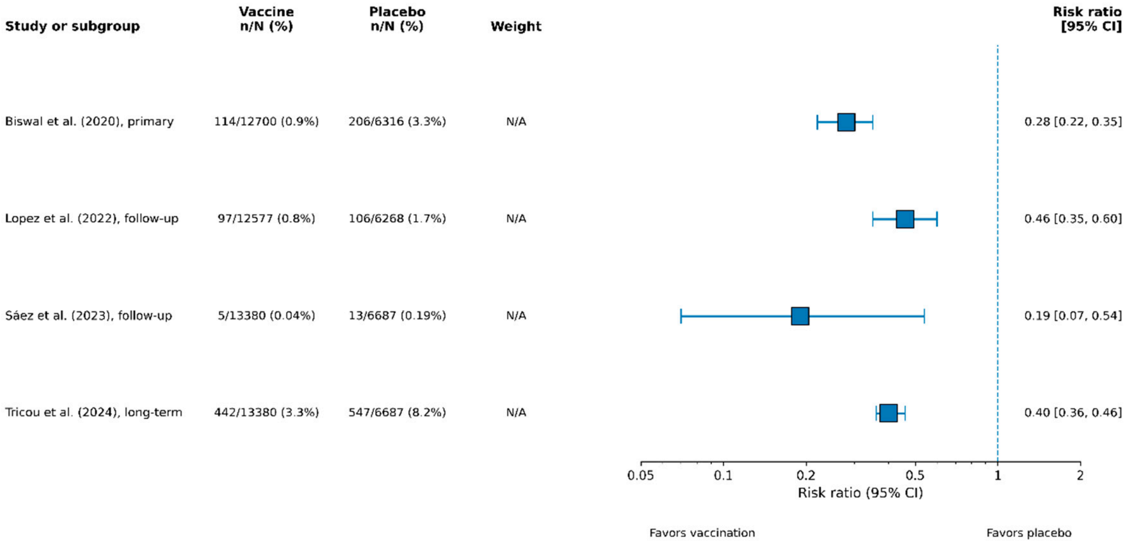

Supplementary Figure S4. TAK-003 efficacy and follow-up estimates against virologically confirmed dengue.

The primary efficacy report and extended follow-up estimates are displayed descriptively. Study weights were not calculated because the publications involved overlapping follow-up of the same trial population and were not pooled as independent evidence. Event counts, denominators, absolute event percentages, risk ratios, and 95% confidence intervals are shown.

Study or subgroup	Vaccine n/N (%)	Placebo n/N (%)	Weight	Risk ratio [95% CI]
**DENV-1**				
Capeding et al. (2014)	51/6548 (0.8%)	50/3210 (1.6%)	48.6%	0.50 [0.34, 0.74]
Villar et al. (2015)	66/12478 (0.5%)	66/6196 (1.1%)	51.4%	0.50 [0.35, 0.70]
**Pooled estimate**	**117/19026 (0.6%)**	**116/9406 (1.2%)**	**100.0%**	**0.50 [0.39, 0.64]**
**DENV-2**				
Capeding et al. (2014)	38/6561 (0.6%)	29/3253 (0.9%)	46.8%	0.65 [0.40, 1.05]
Villar et al. (2015)	58/12495 (0.5%)	50/6219 (0.8%)	53.2%	0.58 [0.40, 0.84]
**Pooled estimate**	**96/19056 (0.5%)**	**79/9472 (0.8%)**	**100.0%**	**0.60 [0.45, 0.81]**
**DENV-3**				
Capeding et al. (2014)	10/6613 (0.15%)	23/3281 (0.7%)	38.0%	0.22 [0.10, 0.46]
Villar et al. (2015)	43/12514 (0.3%)	82/6213 (1.3%)	62.0%	0.26 [0.18, 0.38]
**Pooled estimate**	**53/19127 (0.3%)**	**105/9494 (1.1%)**	**100.0%**	**0.25 [0.18, 0.35]**
**DENV-4**				
Capeding et al. (2014)	17/6605 (0.3%)	34/3265 (1.0%)	49.1%	0.25 [0.14, 0.44]
Villar et al. (2015)	18/12522 (0.1%)	40/6206 (0.6%)	50.9%	0.22 [0.13, 0.39]
**Pooled estimate**	**35/19127 (0.2%)**	**74/9471 (0.8%)**	**100.0%**	**0.23 [0.16, 0.35]**

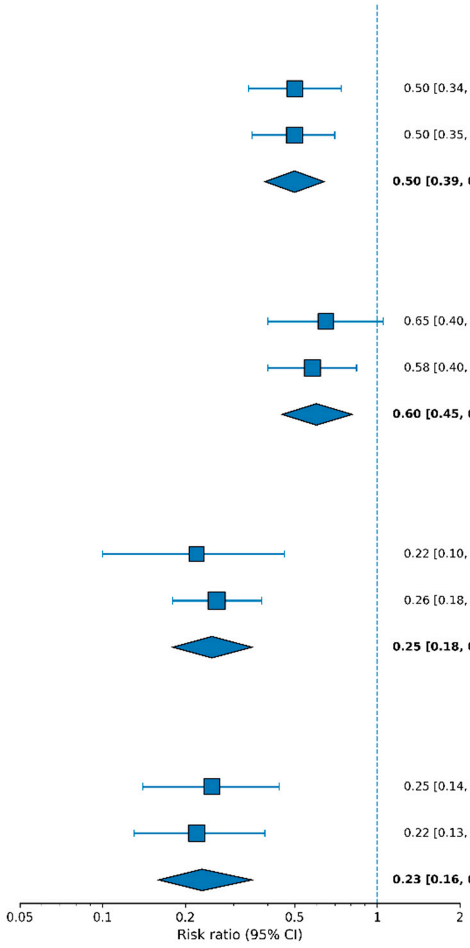

Favors vaccination   Favors placebo

Supplementary Figure S5. CYD-TDV serotype-specific efficacy.

The two independent CYD-TDV trials and their pooled estimate are presented separately for DENV-1, DENV-2, DENV-3, and DENV-4. No overall cross-serotype estimate was calculated because serotype-specific outcomes were correlated within the same participants. Event counts, denominators, absolute event percentages, study weights, risk ratios, and 95% confidence intervals are shown.

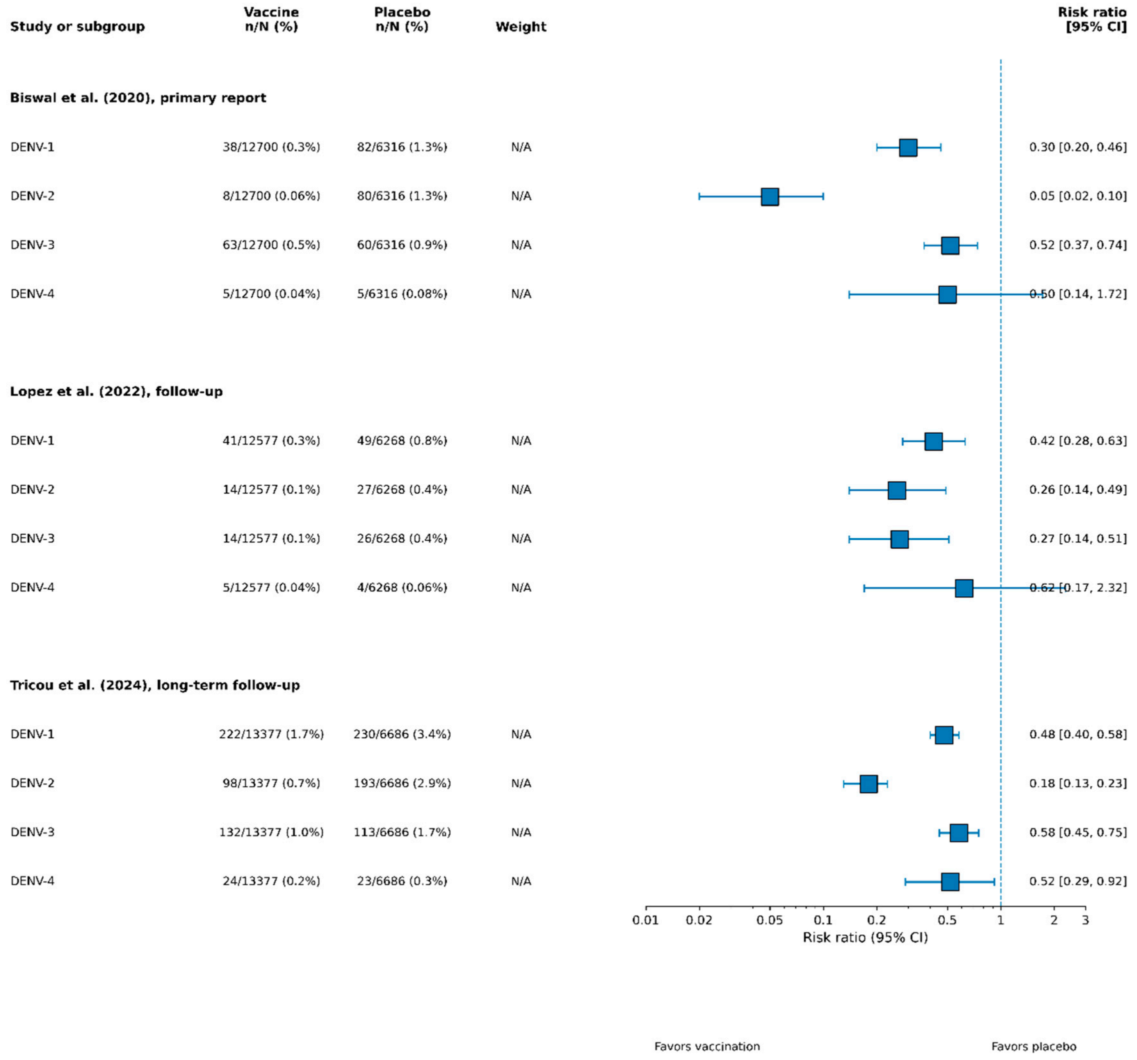

Supplementary Figure S6. TAK-003 serotype-specific efficacy and follow-up estimates.

Estimates are presented separately by report and DENV serotype. Study weights were not calculated because the follow-up publications involved overlapping participants and were displayed descriptively rather than pooled as independent evidence. Event counts, denominators, absolute event percentages, risk ratios, and 95% confidence intervals are shown.

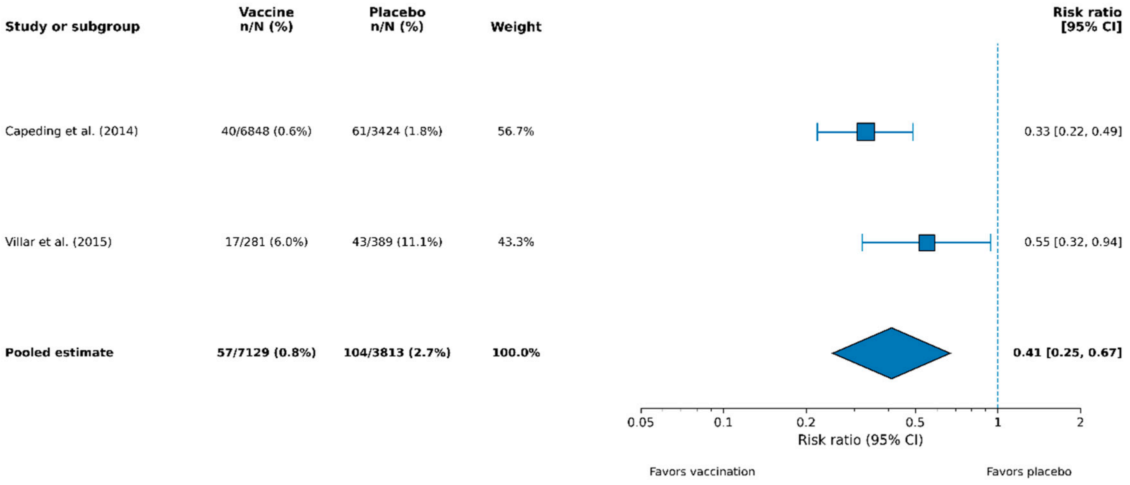

Supplementary Figure S7. CYD-TDV effect on dengue-related hospitalization.

The plot includes two independent randomized efficacy trials and their pooled estimate. Event counts, denominators, absolute event percentages, study weights, risk ratios, and 95% confidence intervals are shown. Risk ratios below 1 favor CYD-TDV.

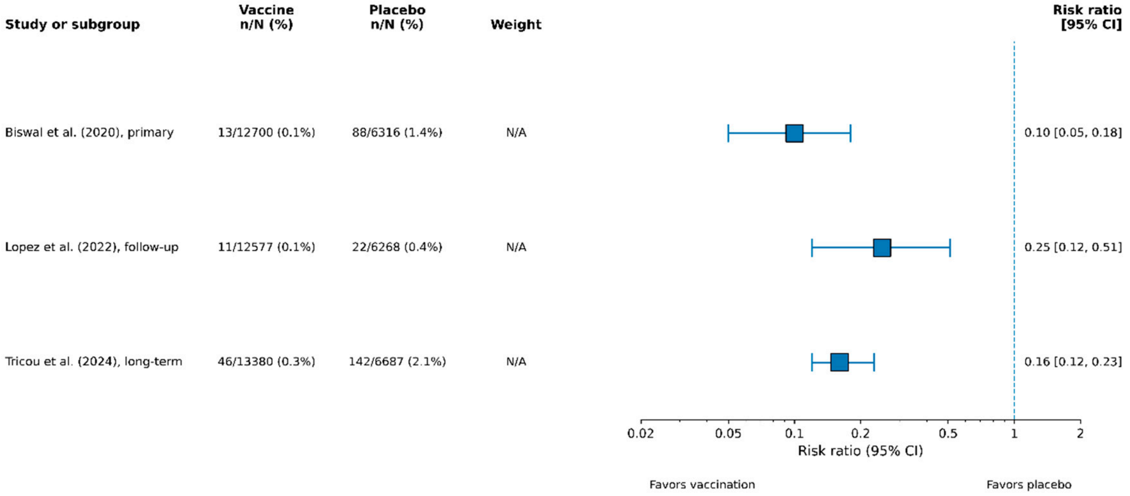

Supplementary Figure S8. TAK-003 effect and follow-up estimates on dengue-related hospitalization.

The primary efficacy report and extended follow-up estimates are displayed descriptively. Study weights were not calculated because the reports represented overlapping follow-up of the same trial population and were not pooled as independent evidence. Event counts, denominators, absolute event percentages, risk ratios, and 95% confidence intervals are shown.
